# Exosomes-mediated Transfer of miR-125a/b in Cell-to-cell Communication: A Novel Mechanism of Genetic Exchange in the Intestinal Microenvironment

**DOI:** 10.7150/thno.41802

**Published:** 2020-06-12

**Authors:** Wei Cheng, Kai Wang, Zhenguo Zhao, Qi Mao, Gang Wang, Qiurong Li, Zheng Fu, Zhiwei Jiang, Jian Wang, Jieshou Li

**Affiliations:** 1 Research Institute of General Surgery, Jinling Hospital, Medical School of Nanjing University, Nanjing, 210000, China.; 2 State Key Laboratory of Pharmaceutical Biotechnology, Jiangsu Engineering Research Center for MicroRNA Biology and Biotechnology, School of Life Sciences, NJU Advanced Institute for Life Sciences, Nanjing University, Nanjing, 210000, China.; 3 Department of General Surgery, Jiangsu Province Hospital of Chinese Medicine, Affiliated Hospital of Nanjing University of Chinese Medicine, Nanjing, 210000, China.; 4 Department of General Surgery, The Affiliated Hospital of Xuzhou Medical University, Xuzhou, Jiangsu, 221000, People's Republic of China.; 5 Department of General Surgery, Jiangyin Hospital Affiliated to Nantong University, Jiangyin, Jiangsu, 214400, People's Republic of China.

**Keywords:** Exosomes, Glucagon-like peptide-2, miR-125a/b, Short bowel syndrome, Proliferation and apoptosis of intestinal epithelial cells

## Abstract

Glucagon-like peptide-2 (GLP-2), a key factor in intestinal rehabilitation therapy of short bowel syndrome (SBS), may require cell-to-cell communication to exert its biological functions. However, understanding of the mechanism remains elusive. Here, we report participation of exosomal miR-125a/b in GLP-2 mediated intestinal epithelial cells-myofibroblasts cross-talk in intestinal microenvironment.

**Methods:** The effects of GLP-2 on the proliferation and apoptosis of intestinal epithelial cells in SBS rat models were evaluated. Exosomes were extracted from residual jejunum tissue of GLP-2 or vehicle treated SBS rats using ultracentrifugation method, and identified by nanoparticle trafficking analysis (NTA), transmission electron microscopy and western blotting. miRNA sequencing combined with qRT-PCR validation were used to identify differentially expressed miRNAs. miRNAs, which might be involved in proliferation and apoptosis of intestinal epithelial cells, were screened and further verified by miRNA functional experiments. Moreover, the proliferation-promoting and anti-apoptosis effects of GLP-2 on intestinal myofibroblasts, which expressing GLP-2 receptor, and whether GLP-2 could influence the content of miRNAs in the derived exosomes were studied. The downstream pathways were explored by miRNA function recovery experiment, luciferase reporter assay, pull down experiment, knockdown and overexpression of target gene and other experiments based on the bioinformatics prediction of miRNA target gene.

**Results:** GLP-2 significantly promoted intestinal growth, facilitated the proliferation of intestinal crypt epithelial cells and inhibited the apoptosis of intestinal villi epithelial cells in type II SBS rats. GLP-2 significantly down-regulated exosomal miR-125a/b both in residual jejunums derived exosomes and in exosomes secreted by GLP-2R positive cells. Exosomal miR-125a/b was responsible for GLP-2 mediated intestinal epithelial cells proliferation promotion and apoptosis attenuation. miR-125a/b inhibited the proliferation and promotes apoptosis of intestinal epithelial cells by suppressing the myeloid cell leukemia-1 (MCL1).

**Conclusions:** miR-125a/b shuttled by intestinal myofibroblasts derived exosomes regulate the proliferation and apoptosis of intestinal epithelial cells. GLP-2 treatment significantly decreases the level of miR-125a/b in the exosomes of intestinal myofibroblasts. miR-125a/b modulates the proliferation and apoptosis of intestinal epithelial cells by targeting the 3'UTR region of MCL1. Hence, this study indicates a novel mechanism of genetic exchange between cells in intestinal microenvironment.

## Introduction

Short bowel syndrome (SBS) is a kind of disease mainly presented as severe malabsorption, malnutrition, diarrhea and electrolyte disorder resulting from massive small bowel resection due to ischemia, Crohn's disease, gastroschisis, intestinal atresia or trauma. SBS is the most common cause of chronic intestinal failure (CIF) in both children and adults 1, 2. Once patients with SBS cannot achieve enteral autonomy through intestinal rehabilitation treatment, patients will develop irreversible CIF and need lifelong supplemental parenteral nutrition (PN) support, which will bring heavy economic burdens to families and society. What's worse, long-term use of PN may lead to life-threatening complications such as sepsis and chronic liver disease 3. The surgical therapy of SBS, such as surgical reconstruction and small bowel transplantation, is associated with high morbidity and mortality 4. Under these circumstances, intestinal rehabilitation treatment aimed to maximize remnant intestinal absorptive capacity and to wean SBS patients themselves off PN, has become the focus and breakthrough point of SBS treatment.

Glucagon-like Peptide 2 (GLP-2) is an intestinotrophic growth factor secreted by enteroendocrine L cells in the distal ileum and proximal colon, but its biological activities mainly work in proximal small intestine 5. GLP-2^1-33^, a 33 amino acid peptide, serves as a therapeutic focus in the current treatment of SBS. GLP-2 can induce intestinal crypt cells proliferation, inhibit enterocyte apoptosis, promote the proliferation of intestinal villi, and thus promoting the expansion of intestinal mucosa and absorption of nutrients 6. GLP-2 exerts its functions through the 7-transmembrane G protein coupled GLP-2 receptor (GLP-2R) and the expression of GLP-2R is largely confined to gastrointestinal tract, with the highest level in the jejunum (i.e. jejunum > duodenum/ileum > colon > stomach) 7. Studies have shown that intestinal crypt epithelial cell and intestinal villus epithelial cells do not express the GLP-2R, while intestinal stromal cell (such as intestinal subepithelial myofibroblasts, ISEMFs) do express GLP-2R. Previous studies indicated that the biological effect of GLP-2 might be realized through cell-to-cell communication between intestinal epithelial cells and GLP-2R positive cells 8, 9. Nonetheless, the underlying mechanism is still fragmentary and incomplete 8.

Exosomes with lipid bilayer structures are 30-150 nm vesicles released by most cells, and are rich of functional contents such as miRNA and protein. miRNAs have been implicated as critical components of exosomes and largely decide the effects of exosomes on cellular communication 10, 11. Several studies have disclosed that many miRNAs are involved in the proliferation, differentiation and apoptosis of intestinal epithelial cells, such as miR-30 12, miR-33, miR-22, miR-25 and miR-145 13. Given the involvement of miRNAs in many physiological and pathological processes, it is likely that they are also involved in intercellular communication between intestinal epithelial cells and GLP-2R positive cells. The ability of exosomes shuttling different kinds of miRNAs would provide additional control and flexibility to this process. To our knowledge, the regulatory effect of miRNA-loaded exosomes on proliferation and apoptosis of intestinal epithelial cells has been rarely studied 14-16. Whether the miRNA-loaded exosomes serve as a downstream paracrine signal mediator regulated by GLP-2R positive intestinal myofibroblasts needs further exploration.

In this study, we demonstrated that GLP-2 regulated the proliferation and apoptosis of intestinal epithelial cells through down-regulating exosomal miR-125a/b secreted by GLP-2R positive intestinal myofibroblasts, thereby promoting the intestinal adaptation after massive intestinal resection. Our work further confirmed that miR-125a/b involved in exosomes regulated the proliferation and apoptosis process by targeting MCL1.

## Methods

Additional Materials and Methods are in the Supplementary Methods.

### Animal experimental protocol

All procedures with animals were approved by the Animal Care and Use Committee of Jinling Hospital and performed in accordance with the “Guide for the Care and Use of Laboratory Animals published by the National Institutes of Health (Eighth Edition)”. Male Sprague-Dawley rats weighing approximately 250-260 g were housed with a 12-h light-dark cycle and were fed a standard laboratory diet with free access to food and water.

**Figure 1:**
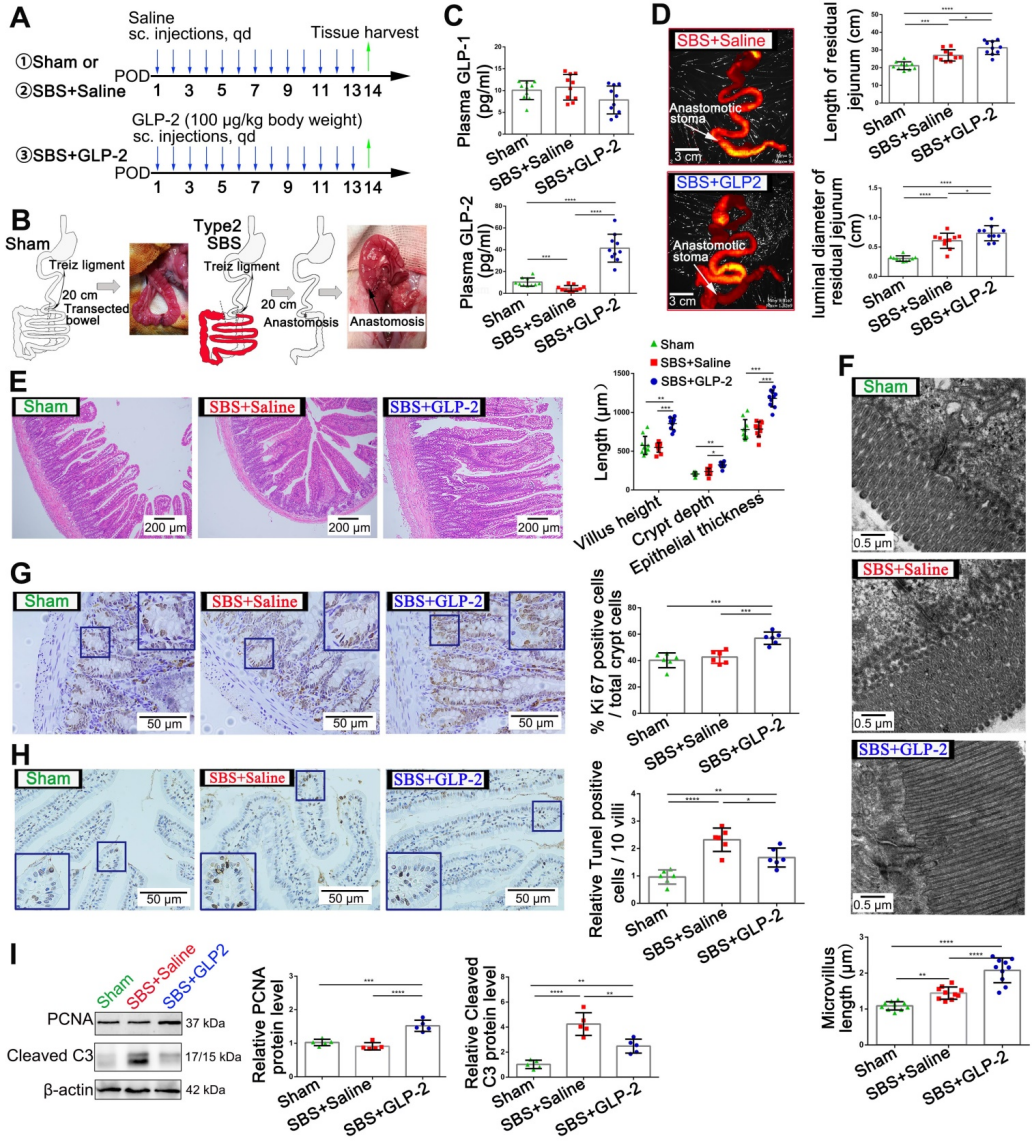
** GLP-2 mediates proliferation and apoptosis of intestinal epithelial cells and promotes intestinal adaptation in SBS rats. (A)** Experimental design for the *in vivo* study. SBS was induced by massive small bowel resection and partial colon resection in male SD rats. Sham-operated rats underwent the same procedure without intestine resection. 100 µg/kg GLP-2 or equal volume of saline was injected subcutaneously once daily for 2 weeks after the surgical procedure. Rats were sacrificed and samples were collected 2 weeks after operation**. (B)** Construction scheme of type 2 SBS and sham model. **(C)** Concentration of GLP-1 and GLP-2 in plasma 2 weeks after operation. **(D)** The length and luminal diameter of residual jejunum 2 weeks following SBS operation. **(E)** H&E staining of remaining jejunum after 2 weeks GLP-2 or saline treatment. Intestinal villus height, intestinal crypt depth and intestinal epithelial thickness were measured and shown at the right panel. **(F)** Length of intestinal microvilli in different groups as measured by electron microscopy. **(G)** Representative images of Ki67 staining and corresponding quantitative analysis of crypt epithelium proliferation in different groups. **(H)** Representative images of TUNEL staining and corresponding quantitative data of villus epithelium apoptosis in different groups. **(I)** Western blot assay for PCNA and cleaved caspase-3 expression in remaining jejunum tissues from SBS and sham-operated rats. N=5-10, *P < 0.05, ** P < 0.01, *** P < 0.001, **** P < 0.0001.

**Figure 2:**
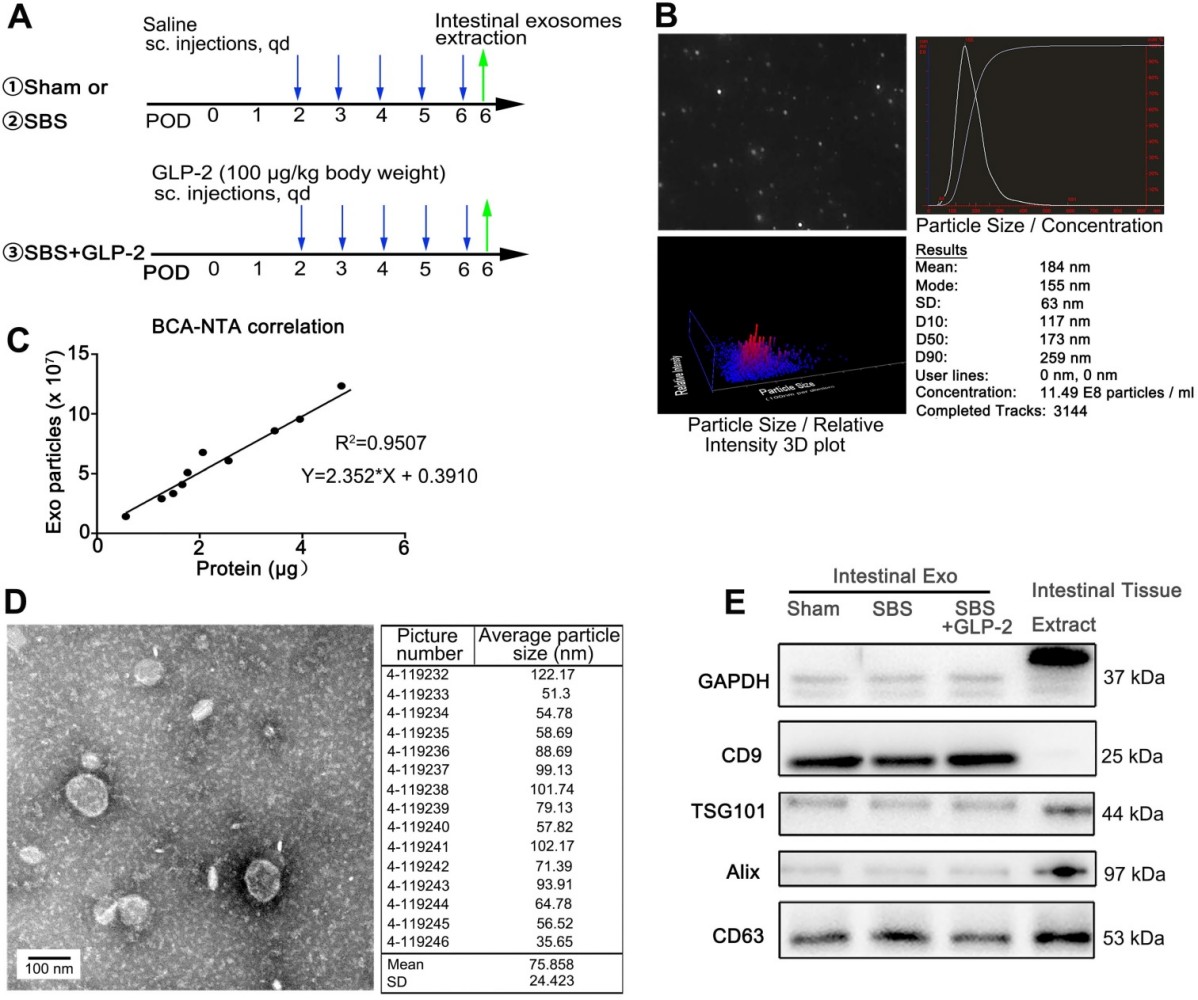
** Characterization of jejunal tissue-derived exosomes. (A)** Schematic illustration of the experimental procedure for intestinal isolation. **(B)** Nanoparticle trafficking analyzed the diameters and concentration of Sham-Exo. **(C)** Correlation between particle number measured by NTA in isolated exosomes and protein weight measured by BCA assay. **(D)** Transmission electron micrograph and particle size distribution of Sham-Exo. **(E)** Representative blots of exosomal marker proteins CD9, TSG101, Alix and CD63 in Sham-Exo, SBS-Exo and GLP2-Exo.

The timeline of the experimental procedures is outlined in Figure 1A and Figure 2A. Animals were fasted for 24 h prior to surgery, with free access to water. All procedures were performed under sodium pentobarbital (40 mg/kg body weight, i.p) using aseptic technique with the aid of surgical telescopes (Designs for Vision, Inc.Ronkonkoma, New York). Animals were randomized to three experimental groups. Group A rats underwent jejunum transection at 20 cm from the ligament of Treitz and re-anastomosis without removal of intestine and were given a saline placebo (Sham group), Group B animals underwent 80% massive small bowel resection, partial colon resection, jejunocolostomy with interrupted 7-0 polydioxanone sutures (Ethicon, Somerville, NJ) and were given a placebo (SBS+Saline group), and Group C animals underwent the same surgical resection as Group B but were treated with recombinant degradation-resistant (Gly^2^)GLP-2^1-33^ (Creative Peptides, USA) by subcutaneous injection with a dose of 100 μg/kg body weight once daily (SBS+GLP-2 group) (shown in Figure 1A-B). The mesenteric blood vessel was ligated with 3-0 polydioxanone suture (Ethicon, Somerville, NJ). Animals were resuscitated with 5 mL warm saline intraperitoneally before abdominal wall closure using 3-0 polydioxanone sutures. Rats were fed a liquid diet (Fresubin, Germany) during the first 24-72 hours after surgery then returned to a chow diet for the remaining postoperative days. Rats in Sham group were given same diet and equal dose of saline administration in line with SBS groups to ensure a matching nutritional intake and control variables.

For antagomir delivery studies, SD rats were intraperitoneally injected with 120 nmol/kg miR-125a antagomir (Ribobio, China) following massive small bowel resection every two days. Control mice were injected with equal dose of NC antagomir. For exosomes administration, SD rats were intraperitoneally injected with 400 μg miR-125a inhibitor or NC inhibitor loaded primary intestinal myofibroblasts exosomes following massive small bowel resection once daily.

### Tissue harvest

Rats were sacrificed on the 6th or 14th postoperative day. The 1 cm jejunum adjacent to the anastomotic site was discarded because of surgery-induced hyperplasia around the anastomosis. The jejunum was split along the anti-mesenteric border, washed with cold phosphate buffer solution (PBS) and dried. Jejunum was harvested for RNA and protein examination (immediately frozen in liquid nitrogen and stored at -80 °C), histological assessment (Fixed with paraformaldehyde or glutaraldehyde) and intestinal exosomes extraction. In addition, jejunum mucosal scrapings were harvested for MCL1 mRNA and protein determination.

### Histology assessment

The length and lumen diameter of residual jejunum from the ligament of Treitz to anastomosis were measured. Tissues were fixed with 4% paraformaldehyde overnight, gradually dehydrated, embedded in paraffin, cut into transverse sections (5 μm thickness) and then stained with hematoxylin and eosin (HE), Ki67 and TUNEL. Quantifications of villus height, crypt depth and epithelial thickness were recorded from 10 representative, well-oriented villus/crypt units, according to HE staining. Crypt cell proliferation was quantified using Ki67 (Abcam, USA) as a marker of active cell division based on previous protocols 17. To assess crypt cell proliferation, we randomly selected six representative, well-oriented crypts and calculated the proliferation index as the ratio of the Ki67-stained nuclei to the total nuclei. To investigate the level of cell apoptosis in the remnant jejunum, TUNEL assays were performed using a commercial kit (*In situ* cell death detection kit, Roche, Germany) according to the manufacturer's instructions. The percentage of TUNEL positive cells in ten well-oriented villi per rat was determined using a light microscope. The TUNEL-positive proportion was expressed as the fold change of the Sham group as described previously 18. In addition, mucosal microvillus structures of each group were detected by transmission electron microscopy (JEM-1400, Japan).

### Exosomes extraction and characterization

Intestinal exosomes were isolated using differential centrifugation based on previously described method with slight modifications 19. Intestinal tissues were isolated and grounded in PBS, and enzymatically digested for 2 h with DMEM medium containing type II collagenase (1 mg/ml; Sigma, USA). All the tissue fragments containing supernatant were centrifuged at 300 g for 10 min at 4 °C. The supernatants were collected and then centrifuged at 3,000 g for 25 min and 10,000 g for 60 min. The supernatants were collected, filtered twice using a 0.22 μm sterile filter (Millipore, USA), and then ultra-centrifuged at 100,000 g for 1 h. Exosomes were washed with 30 ml sterile PBS and centrifuged at 100,000 g for an additional 1 h. The final pellets were resuspended in PBS.

Exosomes secreted by human-derived CCD-18Co cells and rat primary intestinal myofibroblasts were isolated using differential centrifugation based on previously described method 20. When cells reached 70% to 80% confluency, culture medium was replaced with medium that containing 10% exosome-depleted fetal bovine serum and cultured for 48 h. After that, supernatants from cultured CCD-18Co cells or primary intestinal myofibroblasts were centrifuged at 3,000 g for 25 min and then 10,000 g for 1 h at 4 °C. Subsequently, supernatants were filtered through 0.22 μm membrane filters and then centrifuged twice at 100,000 g for 1 h at 4 °C. The exosomes were then resuspended in PBS and then stored at -80 °C for further use.

The morphology of exosomes was observed using transmission electron microscopy (JEM-1011, Japan). After isolation, exosomes were diluted 1:100-600 in filtered PBS and size distribution was measured by NanoSight (NS300; Malvern Inst. Ltd., UK) at 4 °C. Exosomes were quantified by BCA assay (Thermo Fisher, USA) for measurement of total protein. Exosomes were then identified by the exosome marker proteins including CD9, CD63, TSG101 and Alix using western blotting (40 μg exosomal protein per lane). Exosomes were labeled with DiI dye according to the manufacturer's instructions. DiI-labeled exosomes were co-cultured with human intestinal epithelial cell (HIEC6) cells at a final concentration of 20 μg/mL. After 6 h, the cells were washed with PBS and stained with DAPI (Ribobio, China). Finally, the cells were examined and photographed with a confocal microscope (Olympus FV1200, Japan). For *in vivo* uptake, after intraperitoneally injection of DiI-labeled intestinal exosomes in SBS rats, the red-fluorescence intensity of various organs was acquired at 24 h post injection by IVIS spectrum. Absorption efficiency was measured using Living Image 3.1 software.

### miRNA overexpression and knockdown

miRNA 125a/b overexpression and knockdown were achieved by transfecting HIEC6 cells with miRNA mimics or inhibitors, respectively. Synthetic miRNA mimics and inhibitors and scrambled negative control RNAs (NC mimic and inhibitor) were purchased from RioboBio (China). HIEC6 cells were seeded into six-well plates. At 70% confluence, cells were transfected with 200 pmol of miRNA mimics, inhibitors, or corresponding negative control using Lipofectamine 2000 (Invitrogen, USA) according to the manufacturer's instruction.

### Plasmids and RNA interference

Mammalian MCL1 overexpression plasmids and an empty plasmid (control plasmid) were purchased from Genescript (China). siRNA designed to specifically silence MCL1 and scrambled siRNA (control siRNA) were purchased from RioboBio. The sequences of siRNA against MCL1 were as follows: 5′-CGGACTCAACCTCTACTGT-3′. The overexpression plasmids and siRNAs were transfected into HIEC6 cells using Lipofectamine 2000.

### Cell proliferation assay

For cell counting kit-8 (CCK-8) assay, HIEC6 and CCD-18Co cells were plated at 2×10^4^ cells per well in 96-well plates. The cell proliferation index was measured using a Cell Counting Kit-8 (Dojindo, Japan) at 12, 24, 36, 48, and 60 h post-transfection. Absorbance was measured at a wavelength of 450 nm.

For EdU assay, HIEC6 and CCD-18Co cells were seeded in 96-well plates. An EdU assay kit (RiBoBio, China) was used to determine the proliferation rate of the cells. After staining, the cells were captured by confocal microscope.

### Cell apoptosis assay

FITC Annexin V Apoptosis Detection Kit (BD Biosciences) was used in this assay. HIEC6 cells were cultured with exosomes (20 μg/mL) or transfected with miRNA-125a/b mimic/inhibitor, MCL1 plasmid (0.2-1.2 μg/mL) or MCL1 siRNA (50 pmol/mL) for 24 h. CCD-18Co cells and primary intestinal myofibroblasts were treated with GLP-2 (0.1 μg/mL) for 24 h. Then, the cells were treated with 5 μM oxaliplatin for 24 h to induce apoptosis. Cells were trypsinized (without EDTA) into single cells and resuspended with PBS. Next, the cells were stained with FITC-Annexin V and propidium iodide for 15 min in the dark. Samples were finally tested using a flow cytometer (BD Biosciences, USA) and the apoptosis populations were calculated by FlowJo X software.

### Preparation of miR-125a/b mimic loaded-exosomes

To introduce miR-125a/b mimic into exosomes, a modified method of calcium chloride transfection was applied according to previously described method 21. In a 60-mm dish (50-70% confluency) with 5 mL of exosome-free media, 200 pmol FAM-miR-125a/b mimic was mixed with 40 μg exosomes (GLP2-Exo) in PBS then CaCl_2_ was added. The final volume was adjusted to 300 μL using sterile PBS. The mixture was placed on ice for 30 min. After being heat shocked at 42 °C for 60 seconds, the mixture was placed on ice for additional 5 min. To wash the exosomes, 10 ml of PBS was added to the sample, followed by ultracentrifugation at 100,000 g for 60 minutes at 4 °C. Exosomes were then resuspended in PBS. The FAM-miR-125a/b mimic loaded-exosomes were labeled with DiI and cultured with HIEC6 cells as shown in Figure 5A.

### Pull down assay

HIEC6 cells which transfected with biotinylated miR-125a (miR-125a probe) or control probe (Genescript, China) were collected in lysis buffer (20 mM Tris pH7.5, 5 mM MgCl2, 100 mM KCl, 1% NP-40 and 1 U/uL recombinant RNAase inhibitor). After lysis, centrifuges were used to remove cell fragments. DNase I was added to the lysate to digest DNA. After DNA digestion, the lysates were heated at 65 ℃ for 5 minutes, followed by an instant ice bath. Then the lysates were incubated with streptavidin-coated magnetic beads at 4 °C for 4 h with gentle rotation. After incubation, the magnetic beads were washed twice with lysis buffer and RNA bound to magnetic beads was extracted by Trizol for MCL1 and GAPDH qPCR.

### Luciferase reporter assays

Luciferase reporter assays were performed using the luciferase reporter system. The wild-type segment of 3′-UTR of MCL1 contained the predicted miRNA-125a/b target sites (5′-ctcaggga-3′) complementing with the seed sequence of miRNA-125a/b. The mutant segment replaced the target sites by 5′-gagtccct-3′. The wild-type or mutant segments were cloned into the luciferase reporter plasmid to conduct pMIR-MCL1-wt-3′-UTR or pMIR-MCL1-mut-3′-UTR. HIEC6 cells were cultured in 24-well plates, and each well was co-transfected with 200 ng above-mentioned luciferase reporter plasmid, 200 ng β-galactosidase (β-gal) plasmid, and 50 pmol miR-125a mimic, miR-125a inhibitor or scrambled negative control RNAs using Lipofectamine 2000. The β-gal plasmid was served as a transfection efficiency control. Luciferase assay was performed 24 h post transfection using a luciferase assay kit (Promega, USA).

### Statistical analysis

All results are presented as the mean ± SD of at least three independent experiments. Unpaired t-tests (two groups), one-way ANOVA (three or more groups), and Pearson's correlation were analyzed using GraphPad Prism version 6.0 software (GraphPad Software, Inc., USA). Only differences with a P value of less than 0.05 were considered statistically significant.

## Results

### GLP-2 mediates proliferation and apoptosis of intestinal epithelial cells and promotes intestinal adaptation in SBS rats

To investigate the curative effects of GLP-2 on short bowel syndrome, we established the type 2 SBS model in SD rats and 100 μg/kg GLP-2 or vehicle were injected subcutaneously once daily for 2 weeks (Figure 1A-B). Two weeks after SBS operation, the plasma level of GLP-2 in SBS rats was significantly reduced as compared with the sham-operated rats. While the concentration of GLP-2 was significantly increased in GLP-2 treated SBS rats compared to that in Saline-treated SBS rats. No significant difference in plasma glucagon-like peptide-1 (GLP-1) level was found among these three groups (Figure 1C). Then, we observed the intestinal adaptation phenomenon in GLP-2 and saline-treated SBS rats. We found that the extension and expansion of the residual jejunum in GLP-2 treated SBS rats was much better than that in the Saline-treated SBS rats (Figure 1D and Figure S1). Histological assessment of the residual jejunum further confirmed that GLP-2 could significantly promote intestinal villus elongation, intestinal crypt deepening, intestinal microvilli lengthening, intestinal crypt epithelial proliferation (Figure 1E-G) and inhibit intestinal epithelial apoptosis (Figure 1H). Furthermore, the western blot analysis for proliferating cell nuclear antigen (PCNA) and cleaved caspase-3 expression in jejunum tissues also showed that GLP-2 could effectively promote jejunum proliferation and protected jejunum from the apoptosis (Figure 1I), which was consistent with histological analysis. Therefore, GLP-2, as an intestinotrophic growth factor, could maintain the jejunum of SBS rats in a state in which proliferation is significantly enhanced and apoptosis is relatively weakened, thereby facilitating intestinal adaptation.

### Role of intestinal tissue derived exosomes from GLP-2 treated SBS rats in the regulation of proliferation and apoptosis of epithelial cells

As previously reported, intestinal epithelial cells do not express GLP-2 receptor (GLP-2R). Accumulating evidence indicated that GLP-2 exerted its benefit on intestinal epithelial cells via its paracrine effect 8. Exosomes have recently been identified as important mediators of cell-to-cell communication via transferring encapsulated cargoes, such as miRNAs, proteins and bioactive lipids 11, 22, 23. To test the idea that exosomes may be critical for GLP-2 induced proliferative and anti-apoptotic effects in short bowel syndrome, we isolated and purified intestinal exosomes from jejunum of saline treated SBS rats, GLP-2 treated SBS rats and sham-operated rats (called SBS-Exo, GLP2-Exo and Sham-Exo respectively) (Figure 2A). Nanoparticle tracking analysis (NTA) showed the concentration and size distribution of isolated particles, confirming the identity of exosomes (Figure 2B, Figure S2A-B). Moreover, we found that protein weight in these particle fractions measured by BCA assay was strongly correlated with particle number measured by NTA (Figure 2C). Thus, the quantity of exosomes was determined by BCA assay in subsequent study. The morphology of intestine-derived particles was observed directly through transmission electron microscope. The purified vesicles from sham-operated rats, saline treated SBS rats and GLP-2 treated SBS rats showed typical morphology of exosomes with double layer membrane structure and were 75.858±24.423 nm, 76.838±24.515 nm and 63.270±25.647 nm in diameter, respectively (Figure 2D and Figure S2C-D). Additionally, these exosomes contained specific exosomal marker CD9, TSG101, Alix and CD63, while the exclusive marker GAPDH was not detected in intestinal exosomes (Figure 2E).

**Figure 3:**
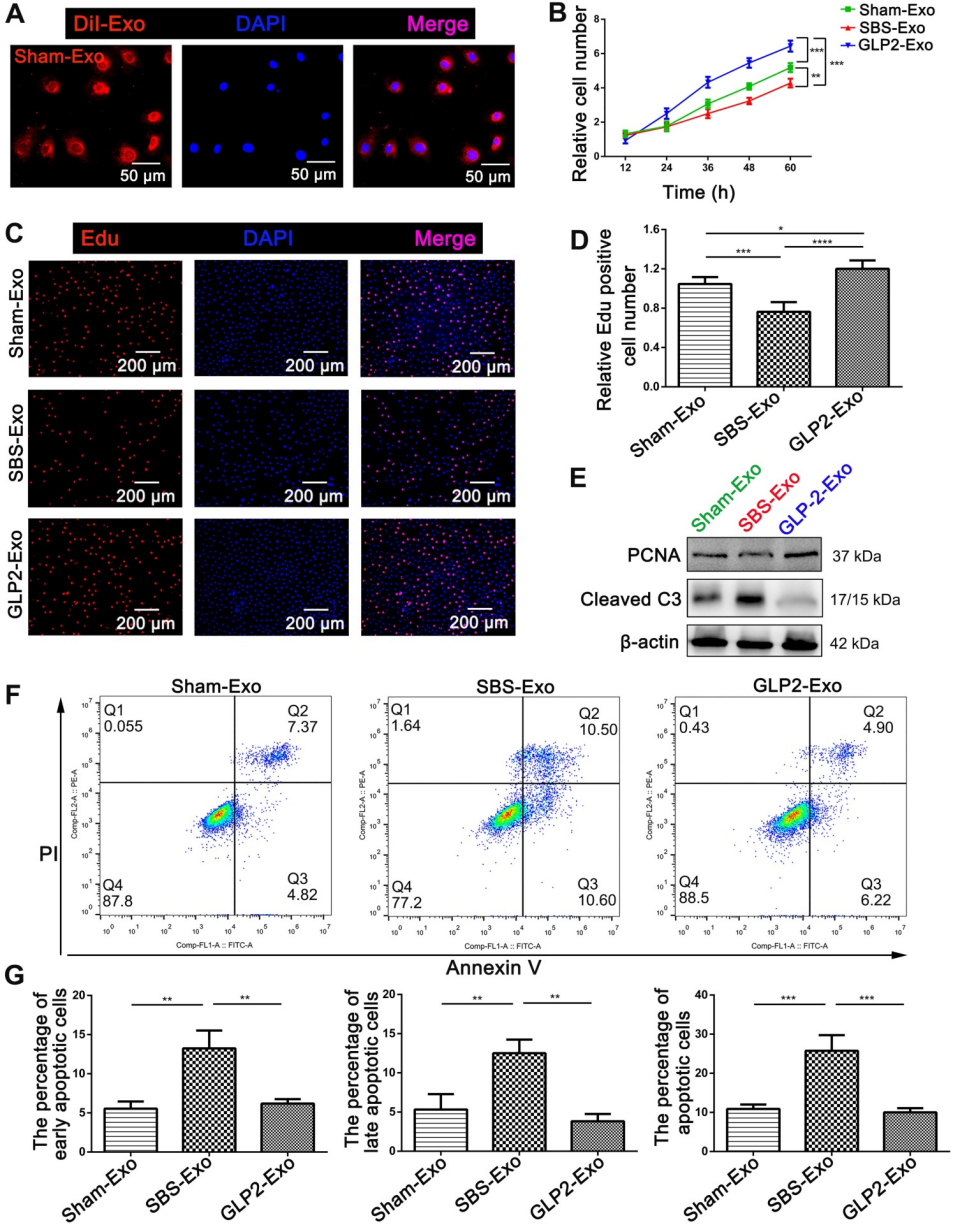
** Intestinal exosomes derived from GLP-2 treated SBS rats exerted proliferative and anti-apoptotic effects on intestinal epithelial cells. (A)** The DiI (red)‐labeled intestinal exosomes (Sham-Exo) were internalized into HIEC6. **(B)** CCK-8 assays were performed 12, 24, 36, 48 and 60 h after adding intestinal exosomes into HIEC6. **(C)** Representative images of EdU staining in HIEC6 after culturing with Sham-Exo, SBS-Exo or GLP2-Exo for 48 h. **(D)** Quantitative assessment of percentage of EdU positive cells in (C). **(E)** Representative blots of PCNA and Cleaved Caspase3 protein levels in HIEC6 after culturing with Sham-Exo, SBS-Exo or GLP2-Exo for 48 h. **(F)** Representative flow cytometry plots showing the percentages of early apoptotic cells (Annexin V^+^/PI^-^) , late apoptotic cells (Annexin V^+^/PI^+^) and total (early + late) apoptotic cells in HIEC6 after culturing with intestinal exosomes. **(G)** Pooled flow cytometry data from (F). N=3, *p < 0.05, **p < 0.01, ***p < 0.001, and ****p < 0.0001.

DiI labeled exosomes were internalized into HIEC6 within six hours (Figure 3A and Figure S3A), indicating intestinal exosomes could be efficiently absorbed by HIEC6. Moreover, *in vivo* uptake tracking showed that intestinal exosomes were robustly accumulated in intestine 24 h after injection, suggesting that intestinal exosomes had intrinsic affinity for intestine (Figure S3B). To investigate the direct effects of intestinal exosomes from different groups on intestinal epithelial cells, we performed CCK-8 and EdU assays to assess the effect of intestinal exosomes on HIEC6 proliferation. Interestingly, compared with Sham-Exo, co-culture of HIEC6 with GLP2-Exo significantly promoted proliferation, whereas SBS-Exo slowed down the proliferation rate (Figure 3B-D). Next, we performed Annexin V/PI dual staining to investigate the effect of intestinal exosomes on HIEC6 apoptosis. Counter to the effect on HIEC6 proliferation, GLP2-Exo significantly reduced apoptosis in HIEC6 as compared with Sham-Exo and SBS-Exo (Figure 3F-G). Western blot and qPCR results further supported the proliferation-promoting and apoptosis-preventing effect of GLP2-Exo (Figure 3E, Figure S4A-C). In addition, qPCR analysis revealed elevated expression of fatty acid transport protein 4 (FATP4) but no changes in insulin-like growth factor 1 receptor (IGF-1R) and sodium-glucose co-transporter 1 (SGLT1) (Figure S4D). Collectively, these data indicated that intestinal exosomes derived from GLP-2 treated SBS rats exerted proliferative and anti-apoptotic effects on intestinal epithelial cells.

### miR-125a/b were key components in intestinal exosomes-regulated proliferation and apoptosis of HIEC6

**Figure 4:**
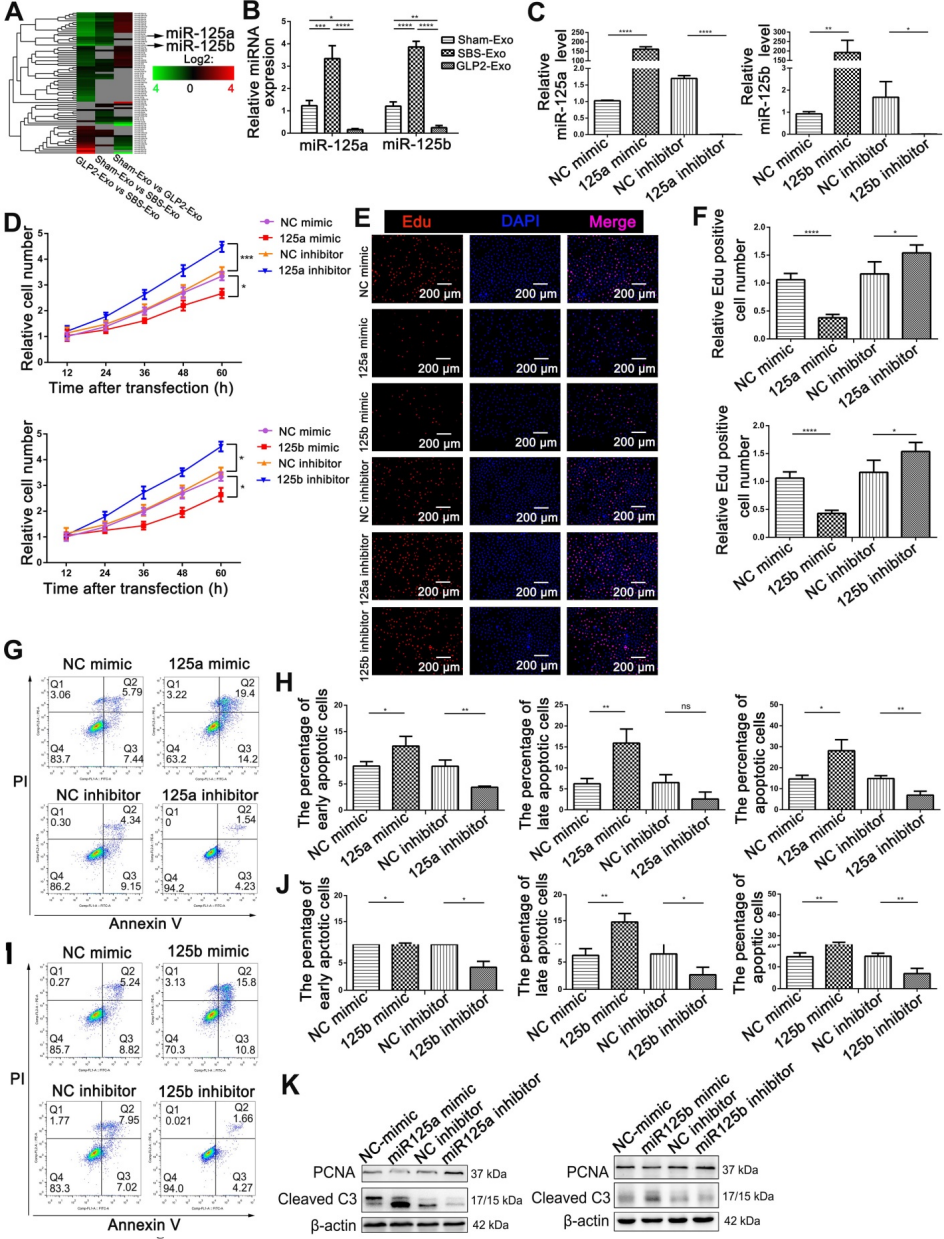
** miR-125a/b were critical in intestinal exosomes mediated proliferation and apoptosis of HIEC6. (A)** miRNA profiling assays were performed in Sham-Exo, SBS-Exo and GLP2-Exo (five exosomes-RNA samples were mixed into one sample for sequencing). Heatmap was generated after supervised hierarchical cluster analysis. **(B)** qPCR analysis of miR-125a/b levels in Sham-Exo, SBS-Exo and GLP2-Exo (N=5). **(C)** qPCR analysis of miR-125a/b levels in HIEC6 cells transfected with NC mimic, miR-125a/b mimic, NC inhibitor or miR-125a/b inhibitor (N=3). **(D)** Cell proliferation assays (CCK-8) were performed 12, 24, 36, 48 and 60 h after the transfection of HIEC6 with equal doses of NC mimic, miR-125a/b mimic, NC inhibitor or miR-125a/b inhibitor (N=3). **(E)** Representative images of EdU staining in HIEC6 transfected with equal doses of NC mimic, miR-125a/b mimic, NC inhibitor or miR-125a/b inhibitor. **(F)** Pooled data of percentage of EdU positive cells in (E) (N=3). **(G-J)** Representative flow cytometry plots and relative quantification of the proportion of early apoptotic cells (Annexin V^+^/PI^-^), late apoptotic cells (Annexin V^+^/PI^+^) and total (early + late) apoptotic cells in HIEC6 after miR-125a/b mimic or inhibitor transfection (N=3). **(K)** Representative blots of PCNA and Cleaved Caspase3 protein levels in HIEC6 transfected with equal doses of NC mimic, miR-125a/b mimic, NC inhibitor or miR-125a/b inhibitor (N=3). *p < 0.05, **p < 0.01, ***p < 0.001, and ****p < 0.0001.

Accumulating evidence suggested that exosomes had an intriguing role on intercellular communication through the exchange of miRNAs between cells 22, 23. Therefore, we determined to screen differences in miRNA contents of Sham-Exo, SBS-Exo and GLP2-Exo using Illumina HiSeq high-throughput sequencing (Figure 4A). A total of 6 up-regulated (fold change>2.0; Figure S5A) miRNAs were detected in SBS-Exo as compared with Sham-Exo, including miR-125b-5p, miR-99a-5p, miR-125a-5p, miR-25-3p, miR-425-5p, miR-30d-5p (in descending order). A total of 25 down-regulated miRNAs (fold change>2.0; Figure S5B) were detected in GLP2-Exo as compared with SBS-Exo, including miR-125b-5p, miR-30d-5p, miR-125a-5p, miR-99a-5p, miR-145-3p, miR-10a-5p (the first six in descending order). Interestingly, miR-125a (ranking the third) and miR-125b (ranking the first) showed consistently increased in SBS-Exo as compared with Sham-Exo, while decreased in GLP2-Exo as compared with SBS-Exo. Same trends of miR-125a/b abundance in Sham-Exo, SBS-Exo and GLP2-Exo were confirmed by q-PCR analysis (Figure 4B). According to miRbase, the sequences of miR-125a/b are completely conserved in human, mouse and rat, and miR-125a/b had the same seed region (nucleotides 2-8^5'→3'^) indicating they might have the same biological functions (Figure S5C). Thus, we hypothesized that miR-125a/b might play an important role in intestinal exosomes-regulated proliferation and apoptosis of HIEC6. To test the hypothesis, we efficiently overexpressed or knocked down the miR-125a/b with miR-125a/b mimics or inhibitors, respectively (Figure 4C). Then, we performed CCK-8 (Figure 4D) and EdU (Figure 4E-F) assays to investigate the effect of miR-125a/b on HIEC6 cell proliferation. As expected, miR-125a/b inhibition activated the proliferation of HIEC6 cells, whereas miR-125a/b overexpression had the opposite effect on cell proliferation. We also performed Annexin V/PI apoptotic assay to investigate the effect of miR-125a/b on HIEC6 apoptosis. Counter to the effect on HIEC6 proliferation, miR-125a/b mimic promoted HIEC6 apoptosis, while miR-125a/b inhibitors reduced HIEC6 apoptosis (Figure 4G-J). Besides, western blot (Figure 4K, Figure S6A-B) and qPCR results (Figure S7A-D) further confirmed that miR-125a/b exerted pro-apoptotic and anti-proliferative effects in HIEC6. Taken together, these results suggested that miR-125a/b stood out as candidates encapsulated in intestinal exosomes probably responsible for proliferation and apoptosis modulation in intestinal epithelial cells.

**Figure 5:**
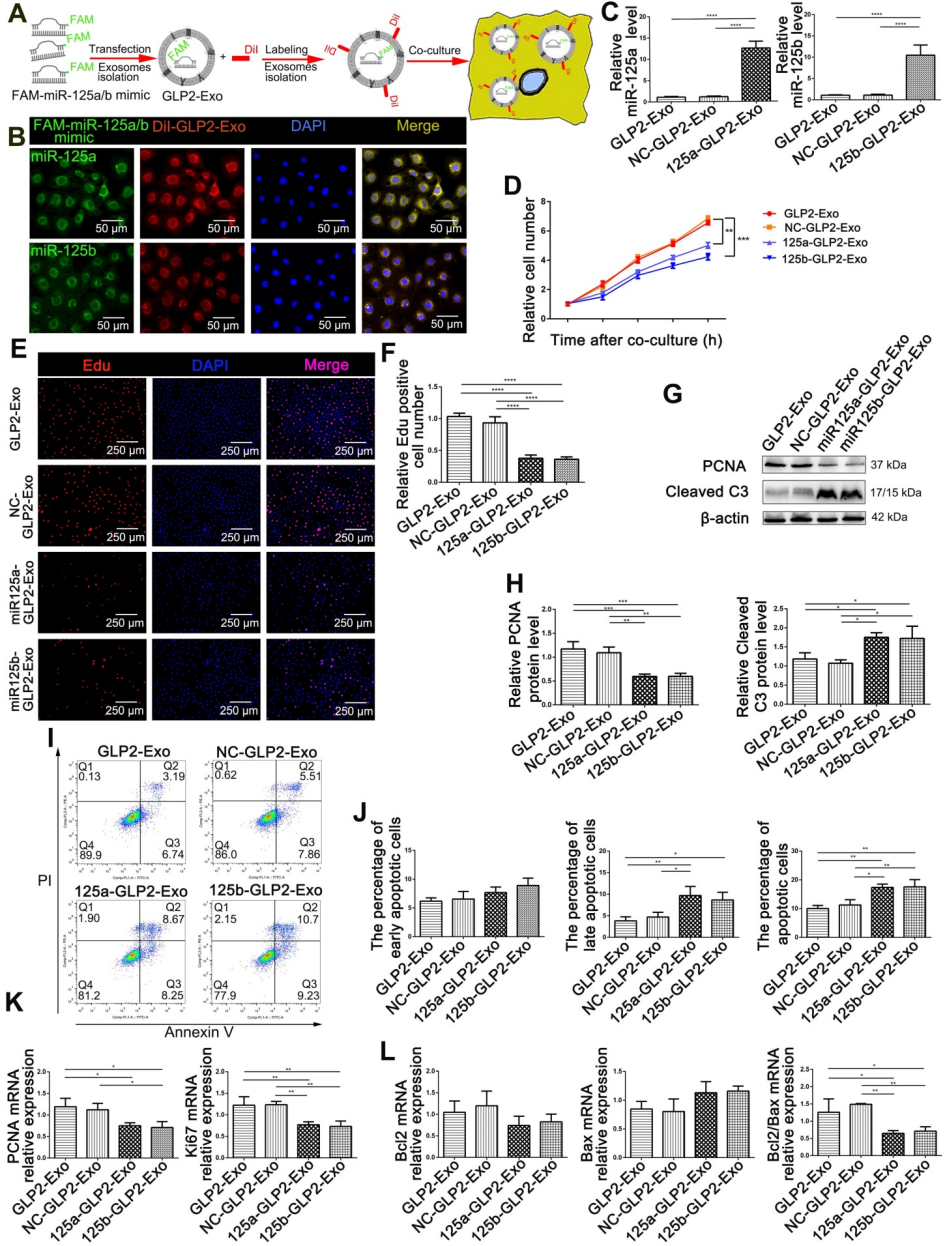
** Encapsulating miR-125a/b mimic into GLP2-Exo blunted its proliferative and anti- apoptosis effects. (A)** Flowchart illustrating the method to introduce miR-125a/b mimic into GLP2-Exo and then internalized by HIEC6. **(B)** FAM-miR-125a/b mimic-loaded GLP2-Exo labeled with DiI were internalized by HIEC6. **(C)** qPCR analysis of miR-125a/b levels in miR-125a/b mimic-loaded GLP2-Exo, NC mimic-loaded GLP2-Exo and GLP2-Exo. **(D)** CCK-8 were performed 12, 24, 36, 48 and 60 h after co-culture of HIEC6 with equal doses of miR-125a/b mimic-loaded GLP2-Exo, NC mimic-loaded GLP2-Exo and GLP2-Exo. **(E)** Representative images of EdU staining in HIEC6 after culturing with miR-125a/b mimic-loaded GLP2-Exo, NC mimic-loaded GLP2-Exo and GLP2-Exo for 48 h. **(F)** Quantitative assessment of percentage of EdU positive cells in (E). **(G)** Representative blots of PCNA and Cleaved Caspase3 protein levels in HIEC6 after culturing with miR-125a/b mimic-loaded GLP2-Exo, NC mimic-loaded GLP2-Exo and GLP2-Exo for 48 h. **(H)** Quantitative analysis of Western blots for PCNA and Cleaved Caspase3 protein levels in (G).** (I-J)** Representative flow cytometry plots and relative quantification of the ratio of early apoptotic cells (Annexin V^+^/PI^-^) , late apoptotic cells (Annexin V^+^/PI^+^) and total (early late) apoptotic cells in HIEC6 after culturing with miR-125a/b mimic-loaded GLP2-Exo, NC mimic-loaded GLP2-Exo and GLP2-Exo for 48 h.** (K)** Gene expression profiles of proliferation marker PCNA and Ki67 in HIEC6 after culturing with miR-125a/b mimic-loaded GLP2-Exo, NC mimic-loaded GLP2-Exo and GLP2-Exo for 48 h. **(L)** Gene expression profiles of anti-apoptotic Bcl-2 and pro-apoptotic Bax in HIEC6 after culturing with miR-125a/b mimic-loaded GLP2-Exo, NC mimic-loaded GLP2-Exo and GLP2-Exo for 48 h. N=3, *p < 0.05, **p < 0.01, ***p < 0.001, and ****p < 0.0001.

To confirm the role of miR-125a/b in the proliferative and anti-apoptotic effect of GLP2-Exo, we used calcium chloride-mediated transfection to encapsulate miR-125a/b mimics or the negative control into the isolated GLP2-Exo (Figure 5A) based on the study of Zhang D and colleagues 21. As expected, GLP2-Exo transfected with miR-125a/b mimic could be efficiently taken up by HIEC6 cells (Figure 5B-C). We then treated HIEC6 with miR-125a/b mimic-loaded GLP2-Exo (miR125a-GLP2-Exo and miR125b-GLP2-Exo), NC mimic-loaded GLP2-Exo (NC-GLP2-Exo) or GLP2-Exo for 48h and then collected the cells for cell proliferation and apoptosis examination. Compared with the GLP2-Exo encapsulated with negative control, miR125a-GLP2-Exo and miR125b-GLP2-Exo significantly inhibited proliferation and promoted apoptosis of HIEC6 (Figure 5D-L), suggesting that the effects of GLP2-Exo were abolished when miR-125a/b mimics were transfected into GLP2-Exo. Collectively, these data indicated that miR‐125a/b was involved in intestinal exosomes‐regulated proliferation and apoptosis of HIEC6.

### GLP-2 mediated proliferation and apoptosis of HIEC6 through decreasing miR-125a/b contents in exosomes secreted by GLP-2R positive intestinal cells

**Figure 6:**
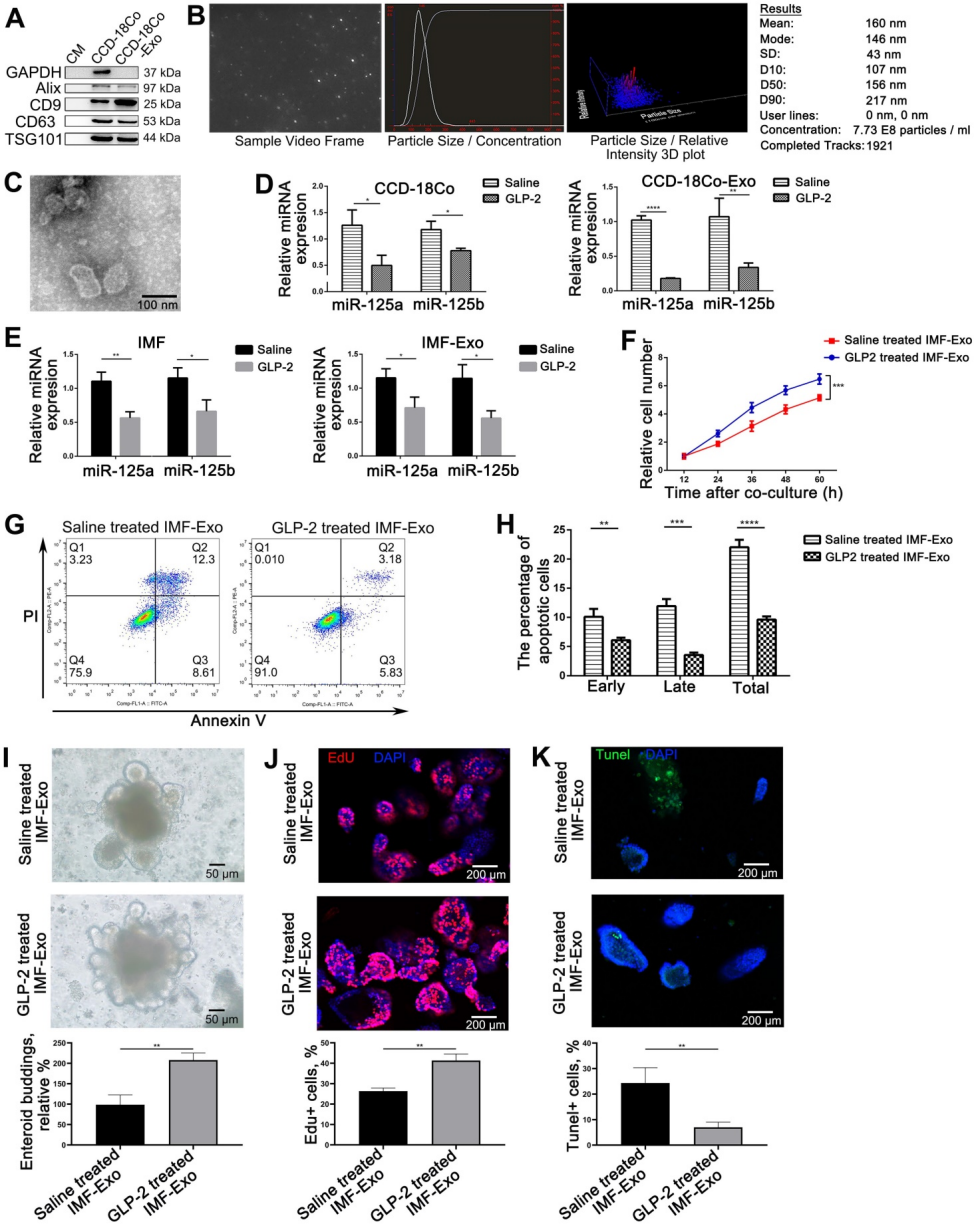
** GLP-2 exerted its proliferative and anti-apoptotic effect on intestinal epithelial through reducing miR-125a/b contents in exosomes derived from GLP-2R positive cells. (A-C)** Identification of CCD-18Co cells derived exosomes by WB (A), NTA (B) and Transmission electron microscopy (C). **(D)** Quantitative analysis of miR-125a/b levels in saline and GLP-2 treated CCD-18Co cells and their exosomes (CCD-18Co-Exo). **(E)** Quantitative analysis of miR-125a/b levels in saline and GLP-2 treated primary intestinal myofibroblasts and their exosomes (IMF-Exo). **(F)** CCK-8 were performed 12, 24, 36, 48 and 60 h after culturing saline or GLP-2 treated IMF-Exo with HIEC6. **(G)** Representative flow cytometry plots showing the percentages of early apoptotic cells (Annexin V^+^/PI^-^), late apoptotic cells (Annexin V^+^/PI^+^) and total (early+late) apoptotic cells in HIEC6 culturing with saline or GLP-2 treated IMF-Exo. **(H)** Quantification of flow cytometry data in (G). **(I)** Representative images and relative budding rate of mice intestinal organoids after culturing with saline or GLP-2 treated IMF-Exo. **(J)** Representative images of EdU staining and relative quantification of mice intestinal organoids under saline or GLP-2 treated IMF-Exo intervention. **(K)** Representative images of TUNEL staining and apoptotic rate of mice intestinal organoids after culturing with saline or GLP-2 treated IMF-Exo. N=3, *p < 0.05, **p < 0.01, ***p < 0.001, and ****p < 0.0001.

Since we observed a decrease in miR-125a/b shuttled by intestinal exosomes under GLP-2 treatment, we decided to focus on the origin of exosomal miRNA-125a decrease. It is well recognized that GLP-2 mainly exerted its effect through binding to specific receptor 8. We therefore asked whether GLP-2 mediated proliferation and apoptosis of intestinal epithelial cells through affecting the miRNA contents of GLP-2R positive cells derived exosomes. CCD-18Co, a kind of intestinal fibroblasts, is the only cell line reported to express GLP-2R 9. We first examined whether GLP-2 could regulate the proliferation and apoptosis of CCD-18Co cells, and found that GLP-2 significantly enhanced the proliferation and prevented apoptosis of CCD-18Co cells (Figure S8). Subsequently, we purified exosomes from the culture supernatants of CCD-18Co using serial differential centrifugation plus ultracentrifugation and then identified CCD-18Co-Exosomes (CCD-18Co-Exo) by WB, NTA and transmission electron (Figure 6A-C). Then, we examined the level of miR-125a/b in CCD-18Co cells and CCD-18Co-Exo by TaqMan probe-based qPCR. As anticipated, GLP-2 dramatically decreased the levels of miR-125a/b both in CCD-18Co cells and CCD-18Co-Exo (Figure 6D) without affecting the packaging and release of exosomes (Figure S9). Same decreasing trends of miR-125a/b were also observed in isolated rat intestinal myofibroblasts (IMF) and their exosomes under GLP-2 intervention (Figure 6E). To further validate that GLP-2 regulates the proliferation and apoptosis of intestinal epithelial cells through decreasing exosomal miR-125a/b secreted by GLP-2R^+^ cells, exosomes from primary IMF (IMF-Exo) with or without GLP-2 intervention were added to HIEC6. CCK8 and Annexin V/PI apoptotic assay demonstrated that exosomes derived from GLP-2 treated IMF significantly promoted proliferation and reduced apoptosis of HIEC6, mimicking the benefits of GLP-2 treated intestinal exosomes (Figure 6F-H). Co-culture of IMF-Exo and organoids further confirmed that GLP-2 treated IMF-Exo significantly enhanced organoid budding and proliferation while inhibiting apoptosis as compared with saline treated IMF-Exo (Figure 6I-K).

To further identify the decrease of miR-125a/b in exosomes secreted by GLP-2R^+^ intestinal myofibroblasts were responsible for GLP-2 regulating proliferation and apoptosis of intestinal epithelial cell, we transfected miR-125a/b mimics or the negative control into the isolated GLP-2 treated IMF-Exo and co-cultured with HIEC6. CCK8 and flow cytometry analysis showed that miR-125a/b mimic loaded GLP2-treated IMF-Exo (miR125a-GLP2 treated IMF-Exo and miR125b-GLP2 treated IMF-Exo) failed to exert the proliferative and anti-apoptotic effects on HIEC6 (Figure S10). Collectively, these data suggested that the proliferative and anti-apoptosis effects of GLP-2 might largely rely on modulating intercellular communication between intestinal epithelial cells and GLP-2R positive cells through reducing miR-125a/b level in exosomes secreted by GLP-2R positive cells.

### miR-125a/b inhibited MCL1 expression at the post-transcriptional level in intestinal epithelial cells

**Figure 7:**
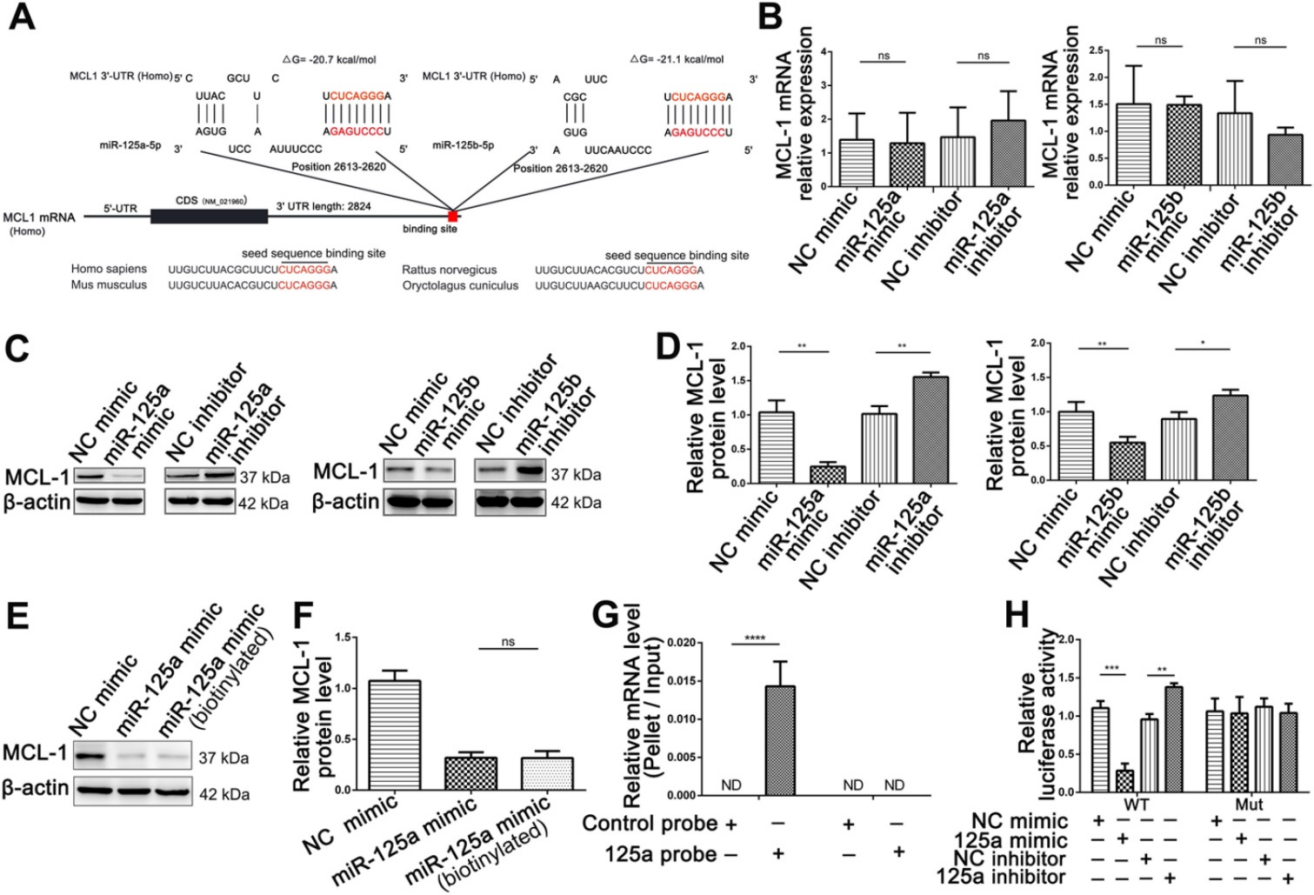
** Identification of MCL1 as a miR-125a/b target. (A)** Schematic description of the hypothetical duplex formed by the interactions between the binding site in the MCL1 3'-UTR and miR-125a/b. The miR-125a/b seed sequence and the seed sequence binding sites in the MCL1 3'-UTR are indicated in red. All nucleotides of the seed sequence of the binding site are conserved in several species, including human, mouse, rat and rabbit. The calculated free energy values of the hybrids are indicated. **(B)** qPCR analysis of MCL1 mRNA levels in HIEC6 cells transfected with miR-125a/b mimic, inhibitor, and scramble negative control. **(C-D)** Representative blots and relative quantification of MCL1 protein levels in HIEC6 cells transfected with miR-125a/b mimic, inhibitor, and scramble negative control. **(E-F)** Representative blots and relative quantification of MCL1 protein levels in HIEC6 cells transfected with NC mimic, miR-125a mimic or biotinylated miR-125a mimic. **(G)** qPCR analysis of MCL1 and GAPDH mRNA levels in HIEC6 after pulling down with control probe or miR-125a probe.** (H)** The relative luciferase activities in HIEC6 transfected with wild type or mutant MCL1 3'-UTR. N=3, *p < 0.05, **p < 0.01, ***p < 0.001, and ****p < 0.0001.

To better understand how miR-125a/b modulates proliferation and apoptosis, we honed in on the target genes for miR-125a/b. Among the potential miR-125a/b target genes predicted by miRanda and TargetScan in human, mouse and rat, we finally focused on MCL1, which had been associated with proliferation and apoptosis process by previous studies 24-26. As shown in Figure 7A, one potential binding site of miR-125a/b was identified at the 3'-UTR of MCL1. To confirm whether MCL1 is a target of miR-125a/b in HIEC6 cells, the expression levels of MCL1 in HIEC6 cells were measured at mRNA and protein level. The protein levels of MCL1 were dramatically decreased in HIEC6 transfected with miR-125a/b mimic and remarkably increased in HIEC6 transfected with miR-125a/b inhibitor, whereas no difference was detected in transcription level (Figure 7B-D). These data indicated that miR-125a/b suppressed the expression of MCL1 within HIEC6 via a post-transcriptional regulation. In order to investigate that miR-125a/b suppressed MCL1 expression through directly binding to the 3'-UTR region of MCL1, we did pull-down assay and luciferase reporter assay. Considering miR-125a and miR-125b have similar sequences, similar expression patterns and almost same binding affinity to MCL1, we focused on miR-125a in further study. Firstly, we performed a biotin-avidin pull-down assay to assess the direct binding of miR-125a to MCL1 mRNA. We designed a miR-125a mimic whose 3' terminal was biotinylated (miR-125a probe). Such modification did not affect the suppression of MCL1 by miR-125a (Figure 7E-F). After transfecting miR-125a probe into HIEC6 for 48 h, we used streptavidin-coated magnetic beads to pull down biotinylated miR-125a and measured the co-precipitated MCL1 mRNA. MCL1 mRNA was only enriched in the pull-down product precipitated by the miR-125a probe and undetectable in the product that was precipitated by control probe (Figure 7G), suggesting that miR-125a directly binds to MCL1 mRNA in HIEC6 cells. As a control, GAPDH mRNA could not be detected in the pull-down product precipitated by the miR-125a probe (Figure 7G), since GAPDH was not a predicted target of miR-125a. Furthermore, To check if it was the predicted seed sequence binding site that caused the miRNA-mRNA interaction, we constructed a firefly reporter plasmid containing a fragment of MCL1 3'-UTR across the conserved miR-125a binding site and then transfected the resulting plasmid into HIEC6 cells along with the miR-125a mimic, miR-125a inhibitor, or corresponding negative control. As expected, overexpression of miR-125a resulted in a significant reduction in luciferase reporter activity compared to cells transfected with the NC mimic, whereas inhibition of miR-125a significantly elevated the luciferase activity in reporter activity compared to cells transfected with NC inhibitor (Figure 7H). Subsequently, we mutated the miR-125a binding site in the MCL1 3'-UTR fragment on the reporter plasmid to abolish miR-125a binding ability. miR-125a overexpression or knockdown no longer affected the mutated reporter activity (Figure 7H), indicating that the binding site strongly contribute to the miRNA-mRNA interactions. Taken together, these results strongly suggested that miR-125a downregulated MCL1 protein level through directly binding to its 3'-UTR and thus induce translational repression.

### MCL1 served as a proliferative and anti-apoptotic protein in intestinal epithelial cells

**Figure 8:**
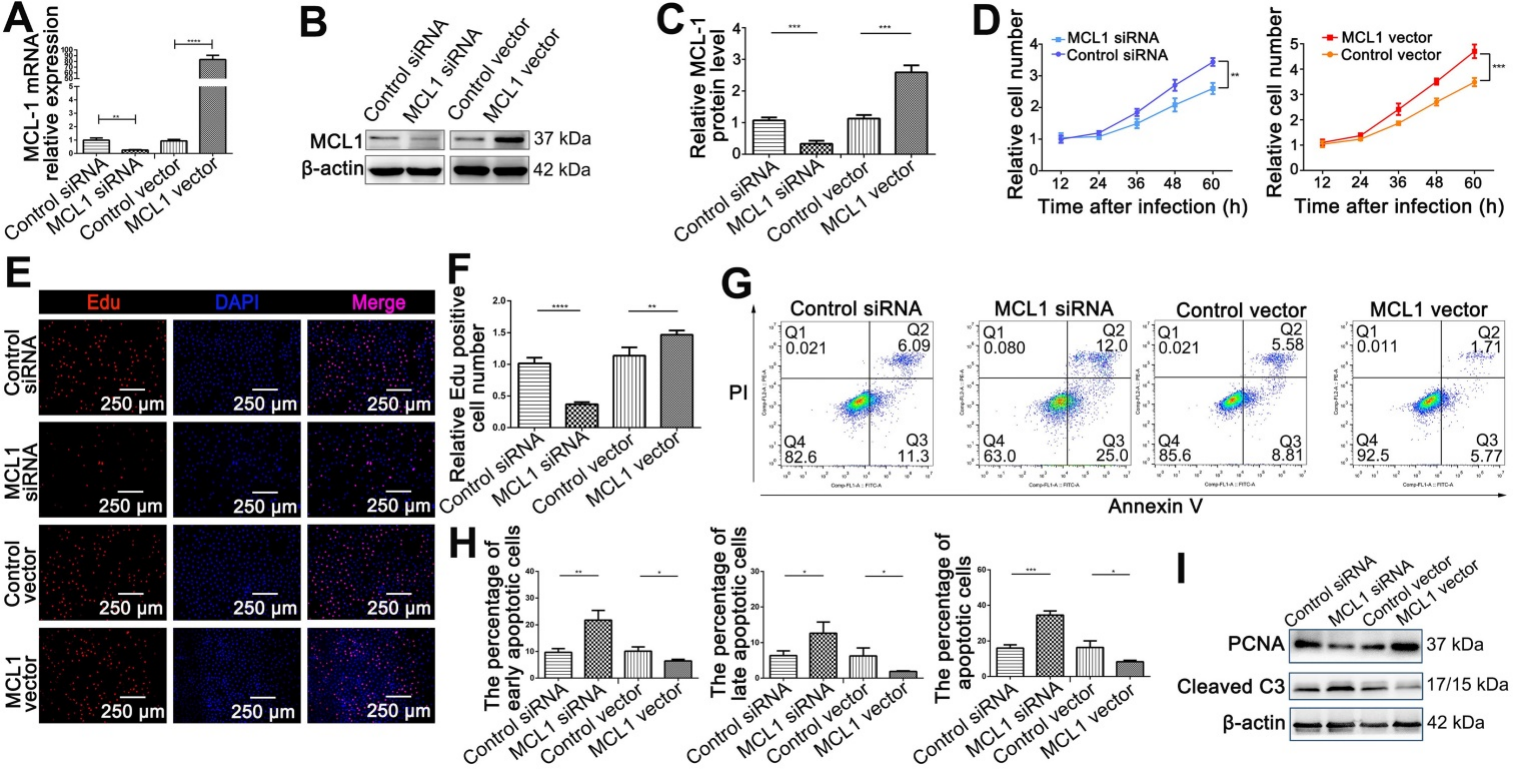
** MCL1 promoted proliferation and inhibited apoptosis of HIEC6 cells. (A)** qPCR analysis of MCL1 mRNA levels in HIEC6 cells transfected with MCL1 plasmid, Control plasmid, MCL1 siRNA, or scrambled Control siRNA. **(B-C)** Representative blots and relative quantification of MCL1 protein in HIEC6 cells transfected with MCL1 plasmid, Control plasmid, MCL1 siRNA, or scrambled Control siRNA. **(D)** CCK-8 were performed 12, 24, 36, 48 and 60 h after the transfection of HIEC6 with MCL1 siRNA, Control siRNA, MCL1 plasmid, or Control plasmid. **(E)** Representative images of EdU staining in HIEC6 transfected with MCL1 plasmid, Control plasmid, MCL1 siRNA, or scrambled Control siRNA. (C) Quantitative assessment of percentage of EdU positive cells in **(F). (G)** Representative flow cytometry plots showing the percentages of early apoptotic cells (Annexin V^+^/PI^-^), late apoptotic cells (Annexin V^+^/PI^+^) and total (early+late) apoptotic cells in HIEC6 transfected with MCL1 plasmid, Control plasmid, MCL1 siRNA, or scrambled Control siRNA. **(H)** Pooled flow cytometry data from (G). **(I)** Representative blots of PCNA and Cleaved C3 protein levels in HIEC6 transfected with MCL1 siRNA, Control siRNA, MCL1 plasmid, or Control plasmid. N=3, *p < 0.05, **p < 0.01, ***p < 0.001, and ****p < 0.0001.

We therefore investigated the exact contribution of MCL1 on HIEC6 proliferation and apoptosis. MCL1 overexpression plasmid (MCL1 vector) and small interfering RNA (MCL1 siRNA) were used to enhance or silence MCL1 expression, respectively (Figure 8A-C). We performed CCK-8 and EdU assays to investigate the effect of MCL1 on HIEC6 proliferation. Results showed that MCL1 overexpression promoted the proliferation, whereas MCL1 knockdown generated an opposite effect (Figure 8D-F). Then, we performed Annexin V/PI dual staining which showed that MCL1 overexpression decreased HIEC6 apoptosis, while MCL1 knockdown increased HIEC6 apoptosis (Figure 8G-H). Western blot (Figure 8I and Figure S11A) and qPCR (Figure S11B-C) further supported the conclusion. The above-mentioned results indicated that MCL1and miR-125a/b had contrary effects on HIEC6 proliferation and apoptosis.

### miR-125a attenuated HIEC6 cell proliferation and promoted apoptosis by targeting MCL1

**Figure 9:**
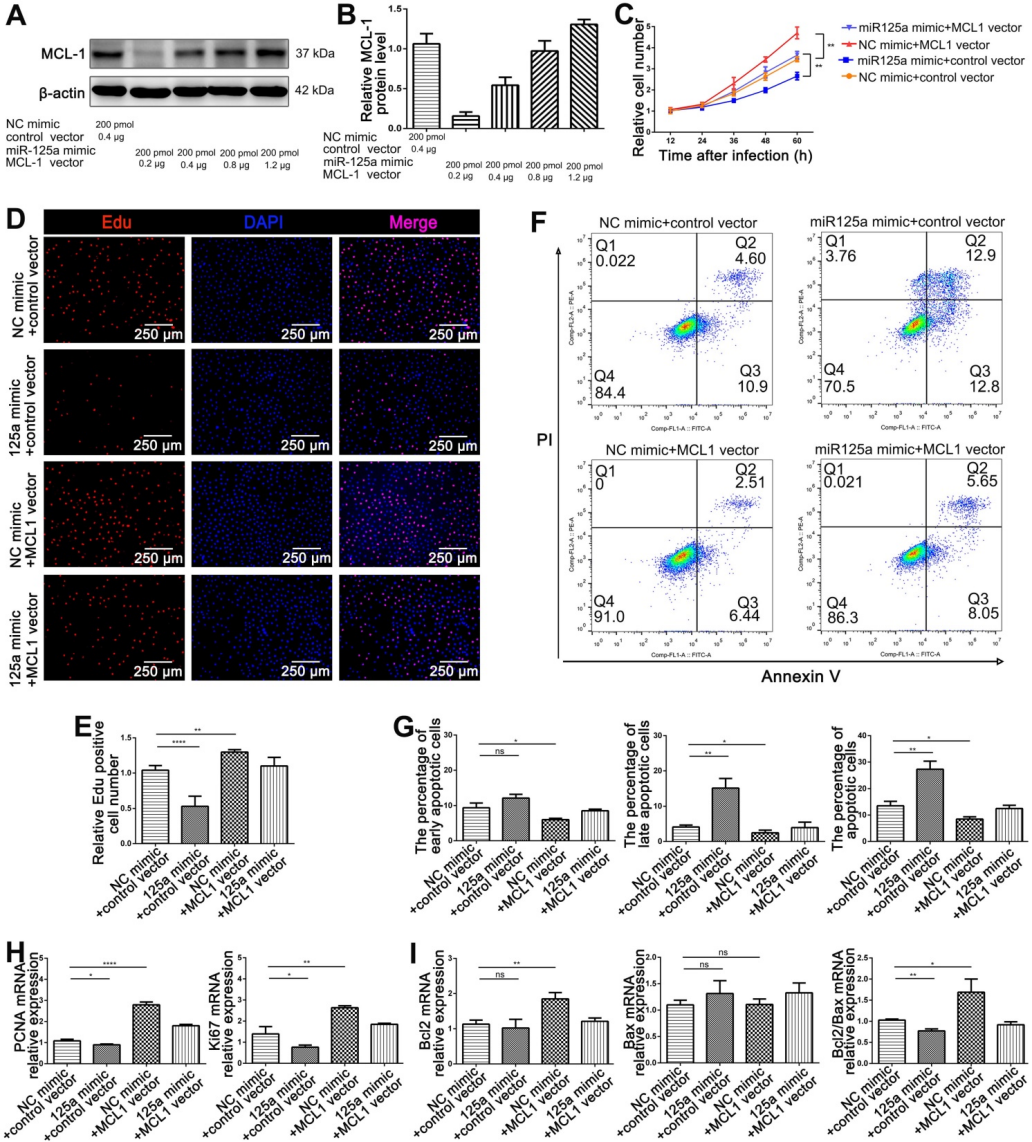
** miR-125a inhibited HIEC6 cell proliferation and promoted apoptosis by targeting MCL1. (A-B)** Pilot study to determine the dosage of MCL1 vector to rescue miR-125a mimic induced MCL1 suppression. **(C)** CCK-8 were performed 12, 24, 36, 48 and 60 h after the co-transfection of HIEC6 with NC mimic+control vector, miR-125a mimic+control vector, NC mimic +MCL1 vector, miR-125a mimic+MCL1 vector. **(D)** Representative images of EdU staining in HIEC6 cells transfected with NC mimic+control vector, miR-125a mimic+control vector, NC mimic +MCL1 vector, miR-125a mimic+MCL1 vector. **(E)** Quantitative analysis of percentage of EdU positive cells in (D). **(F-G)** Representative flow cytometry plots and relative quantification of the ratio of early apoptotic cells (Annexin V^+^/PI^-^), late apoptotic cells (Annexin V^+^/PI^+^) and total (early late) apoptotic cells in HIEC6 transfected with NC mimic+control vector, miR-125a mimic+control vector, NC mimic +MCL1 vector, miR-125a mimic+MCL1 vector. **(H)** Gene expression profiles of proliferation marker PCNA and Ki67 mRNA levels in HIEC6 transfected with NC mimic+control vector, miR-125a mimic+control vector, NC mimic +MCL1 vector, miR-125a mimic+MCL1 vector. **(I)** Gene expression profiles of anti-apoptotic Bcl-2 and pro-apoptotic Bax mRNA levels in HIEC6 cells transfected with NC mimic+control vector, miR-125a mimic+control vector, NC mimic +MCL1 vector, miR-125a mimic+MCL1 vector. N=3, *p < 0.05, **p < 0.01, ***p < 0.001, and ****p < 0.0001.

As elucidated above, miR-125a and MCL1 have opposite biological functions on the proliferation and apoptosis of HIEC6. Since a single miRNA has multiple target genes 27, it is necessary to determine whether the effect of miR-125a on HIEC6 cell proliferation and apoptosis is derived from miR-125a-mediated MCL1 suppression. We therefore investigated the exact contribution of the miR-125a-MCL1 axis on HIEC6 cell proliferation and apoptosis. Firstly, preliminary experiment found that the suppression of 200 pmol miR-125a on MCL1 protein expression could be reversed by 0.8-1.2 μg MCL1 plasmid (Figure 9A-B). We then investigated whether rescuing miR-125a-mediated-MCL1 suppression with 1.0 μg MCL1 overexpression plasmid could reverse the anti-proliferative and pro-apoptotic effect of miR-125a on HIEC6 cells. Results of CCK8 and EdU assays indicated that restoration of MCL1 expression could reverse the negative effect of miR-125a on cell proliferation (Figure 9C-E). In addition, Annexin V/PI flow cytometry results showed that HIEC6 apoptosis triggered by miR-125a was rescued by transfection of MCL1 overexpression plasmid (Figure 9F-G). To validate the above results, we performed qPCR analysis and got a consistent result. (Figure 9H-I). All of these results suggested that miR-125a exerted its effects on proliferation and apoptosis in HIEC6 through silencing MCL1.

### The decrease of exosomal miR-125a/b secreted by intestinal myofibroblasts under GLP-2 treatment modulated the proliferation and apoptosis of intestinal epithelial cells *in vivo* by targeting MCL1

**Figure 10:**
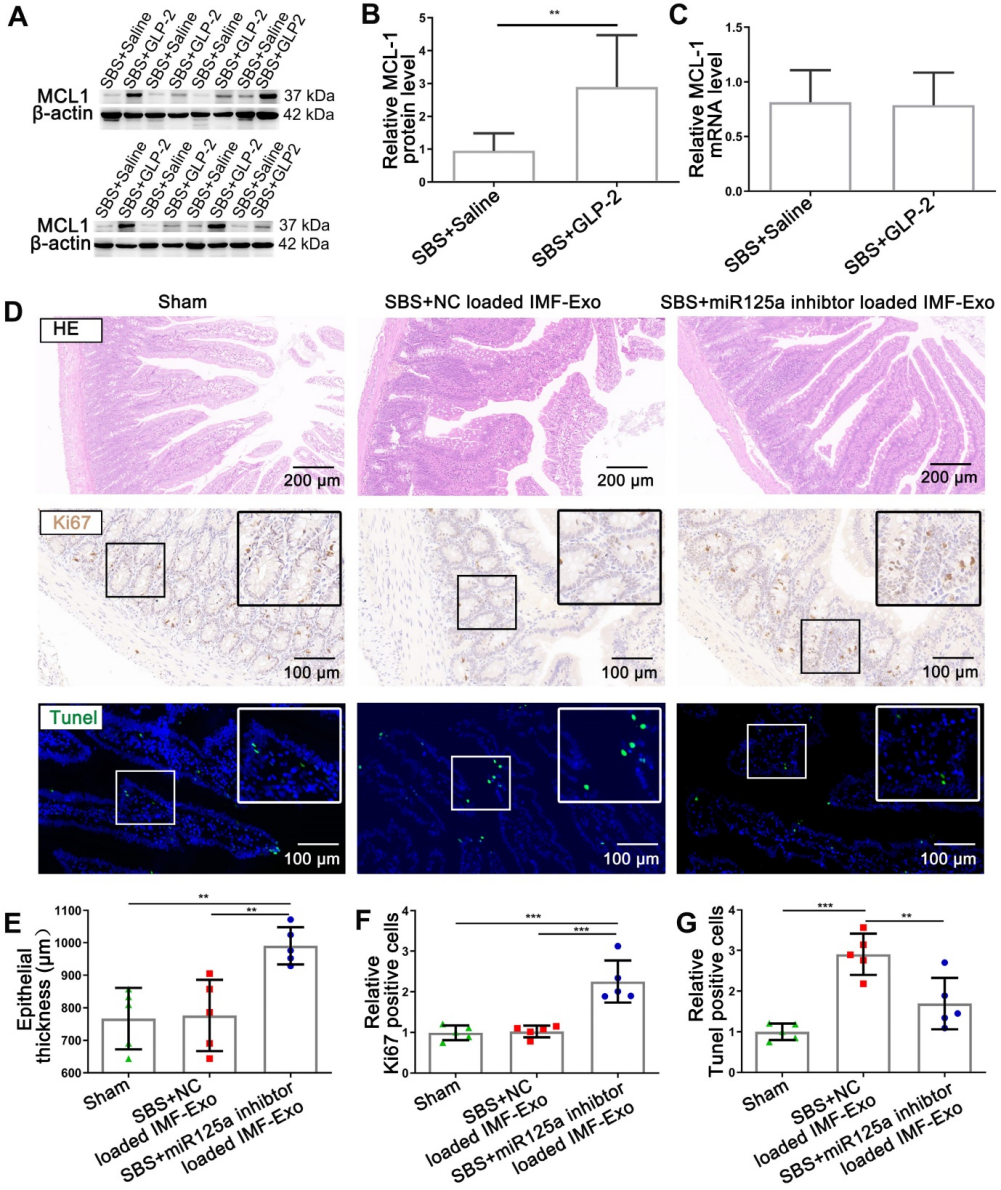
** GLP-2 exerted its intestinotrophic effect by decreasing exosomal miR-125a/b and thus upregulating MCL1 expression *in vivo*. (A-B)** Representative blots and relative quantification of MCL1 protein level in jejunum mucosa of SBS rats treated with GLP-2 or Saline. **(C)** qPCR analysis of MCL1 mRNA levels in jejunum mucosa of SBS rats treated with GLP-2 or Saline. **(D)** Representative images of H&E staining, Ki67 staining and TUNEL staining of remaining jejunum in Sham, SBS+NC loaded IMF-Exo and SBS+miR-125a loaded IMF-Exo rats. **(E)** Quantification of intestinal epithelial thickness in (D). **(F)** Quantitative analysis of percentage of Ki67 positive cells in (D). **(G)** Quantitative analysis of percentage of TUNEL positive cells in (D). N=5-8, **p < 0.01, ***p < 0.001.

To determine the relationship between MCL1 expression and GLP-2 treatment *in vivo*, we assessed protein and mRNA levels in intestinal mucosa of SBS rats with or without GLP-2 treatment. Results showed that MCL1 protein levels in the jejunum mucosa of GLP-2 treated SBS rats were significantly up-regulated than that of saline treated SBS rats (Figure 10A-B), whereas MCL1 mRNA levels exhibited no significant difference (Figure 10C). We then analyzed the relevance of MCL1 protein and mRNA levels using Pearson correlation and found that there was little correlation between the two levels (Figure S12A). The inconsistency between MCL1 protein and mRNA expression in jejunum under GLP-2 treatment supported that the inhibition of MCL1 expression occurred at the post-transcriptional level. Furthermore, Pearson correlation analysis implicated that MCL1 protein levels were strongly correlated with plasma GLP-2 concentrations (Figure S12B) and negatively correlated with exosomal miR-125a/b (Figure S12C-D).

To further validate that reduction of miR-125a/b in exosomes secreted by intestinal myofibroblasts was responsible for GLP-2 mediated intestinal adaptation, miR-125a inhibitor-loaded primary intestinal myofibroblasts exosomes or NC inhibitor-loaded primary intestinal myofibroblasts exosomes were delivered intraperitoneally to SBS rats and the histology of small intestine was studied. Histological assessment of jejunum demonstrated that miR-125a inhibitor-loaded IMF-Exo could significantly promote the growth of intestinal epithelial and inhibit intestinal epithelial apoptosis, recapitulating the effects of GLP-2 *in vivo* (Figure 10D-G). Moreover, SBS rats injected with miR-125a antagomir exhibited fasting intestinal epithelial proliferation and less apoptosis compared with NC antagomir treated SBS rats (Figure S13A-D). Besides, the protein levels of MCL1 were significantly increased in jejunum mucosa of miR-125a antagomir treated SBS rats (Figure S13E-G). Taken together, these results suggested that GLP-2 exerted its intestinotrophic functions by decreasing exosomal miR-125a/b and thus upregulating MCL1 expression *in vivo*.

## Discussion

SBS is the main cause of chronic intestinal failure in children and adults with high morbidity and mortality 28. Over the past decade, the intestinotrophic potential of GLP-2, secreted by L cells in terminal ileum and colon, has generated new hope for improving intestinal adaption in SBS 29. In this study, we further confirmed the proliferative and anti-apoptotic effects of GLP-2 on intestinal epithelial in SBS rats. It is well recognized that GLP-2 mainly exerted its effect through binding to specific receptor 8. Interestingly, previous studies showed that GLP-2 receptors (GLP-2R) were mainly expressed in intestinal stromal cells (such as IMFs) instead of intestinal epithelial cells in the jejunum of mice, rat, and human 8. The underlying mechanism of GLP-2 in the regulation of intestinal epithelial cells remains unclear 8. Recent striking studies disclosed that the apoptosis, proliferation and differentiation of intestinal stem cells were mainly regulated by the intermediary factors from nearby subepithelial stromal cells called niche cells, which sheded light on the understanding of cell-to-cell communication in intestinal microenvironment 30-32. Exosomes have recently been identified as important mediators of intercellular communication via transferring the encapsulated cargoes, such as bioactive lipids, miRNAs, DNA and proteins 23, 33, 34. However, whether exosomes regulate the self-renewal of intestinal epithelium in the intestinal microenvironment remains unknown. In this study we extracted and identified intestinal exosomes from SBS rats under GLP-2 or vehicle treatment and then confirmed that intestinal exosomes could be internalized by intestinal epithelial cells and regulate the proliferation and apoptosis of intestinal epithelial cells. Particularly, GLP-2 treated intestinal exosomes exerted significant proliferation-promoting and apoptosis-preventing effects, suggesting that GLP-2 improved the “efficacy” of intestinal exosomes.

miRNAs have been recognized as important components of exosomes and largely decide the effects of exosomes on recipient cells 20, 35. Using next-generation sequencing, we found 6 up-regulated miRNAs in SBS-Exo compared with Sham-Exo and 25 down-regulated miRNAs in GLP2-Exo compared with SBS-Exo. Among these miRNAs, we identified miR-125a and miR-125b as our top candidates due to the most significant fold change and previous studies addressing its modulation in proliferation and apoptosis 26, 36, 37. Additionally, in our study, we found that miR-125a and miR-125b possessed almost the same biological functions. miR-125a/b overexpression exhibited decreased HIEC6 proliferation and increased apoptosis, whereas miR-125a/b knock-down exhibited increased HIEC6 proliferation and decreased apoptosis, indicating that miR-125a/b might play an important role in intestinal exosomes-regulated proliferation and apoptosis of HIEC6. Since exosomes contain a variety of miRNAs and proteins, it is necessary to determine whether the effect of GLP2-Exo on HIEC6 proliferation and apoptosis is derived from decreasing levels of miR-125a/b. We thus investigated whether restoration of miR-125a/b in GLP2-Exo could blunt the effects of GLP2-Exo. As expected, our study demonstrated that increasing miR-125a/b in GLP2-Exo blunted the proliferative and anti-apoptotic effects of GLP2-Exo on HIEC6 cells. Taken together, these results provided compelling evidence that exosomal miR-125a/b, might play an important role in GLP2-regulated proliferation and apoptosis of intestinal epithelial cells. This is the first report to our knowledge that demonstrates altered functional miRNA level in intestinal exosomes triggered by GLP-2 in SBS model.

Next, we investigated the origins of the GLP2-induced decrease of intestinal exosomal miR-125a/b. Previous study disclosed that the biological responses to GLP-2 were mediated via the GLP-2 receptor (GLP-2R), which is a member of the glucagon/secretin G protein-coupled receptor (GPCR) superfamily 38. Given the GLP-2R expression primarily on intestinal myofibroblasts and lack of GLP-2R expression on intestinal epithelial cells 9, we hypothesized that GLP-2 could down-regulate miR-125a/b expression in intestinal myofibroblasts and thus reducing the relative miRNA-125a/b encapsulated in exosomes. Our data confirmed that the level of miR-125a/b in GLP-2R positive cell line (CCD18-Co), primary intestinal myofibroblasts and their secreted exosomes was significantly reduced under GLP-2 treatment. Meanwhile, GLP-2 treated IMF-Exo recapitulated the proliferation-promoting and apoptosis-preventing effects of GLP-2, while transferring miR-125a/b into GLP-2 treated IMF-Exo blunted these effects. Collectively, these findings indicated that the GLP-2 mediate intestinal epithelial growth was at least partially mediated by the decreasing IMF exosomal transfer of miR-125 a/b. As for the underlying mechanism of miR-125a/b downregulation in GLP-2 treated intestinal myofibroblasts, former publications presented that epidermal growth factor (EGF) could suppress miR-125a/b transcription in tumor cells 39, 40. Moreover, GLP-2 has been reported to promote EGF expression 41, indicating that GLP-2 may decrease exosomal miR-125a/b through upregulation of EGF. Further studies are needed to explore the relationship between upregulation of EGF and decrease of exosomal miR-125a/b in intestinal myofibroblasts.

miRNAs act as endogenous suppressors of gene expression by binding to the 3'-untranslated region (3'-UTR) of target mRNAs to induce translational repression (detected as changes in protein levels without changes in mRNA) or mRNA cleavage (manifested as reductions in both protein and mRNA levels) 42. According to the bioinformatics analysis and previous publications 24, 25, we identified MCL1 as potential target of miR-125a/b in the regulation of proliferation and apoptosis of intestinal epithelial cells. In this study, we experimentally validated miR-125a/b inhibited MCL1 expression by directly targeting the 3′-UTR of MCL1 mRNA and thus inducing translational repression. Subsequently, we revealed the critical effects of miR-125a/b-driven suppression of MCL1 on the pro-apoptotic and anti-proliferative effects in HIEC6 cells. Despite the compelling evidence pinpointing MCL1 as a likely downstream effector in the modulation of intestinal epithelial growth; it is clear that miRNA simultaneously regulate multiple targets. The modulation of other targets may also influence the process of intestinal adaptation, which needs further investigation.

Finally, we examined the correlation of MCL1 expression with plasma GLP-2 concentrations and exosomal miR-125a/b level in SBS model. Exosomal miR-125a/b functions as a key regulator and a bridge to link upstream stimulation (such as intestinotrophic GLP-2) and downstream effector (such as MCL1). To further clarify GLP-2 regulated intestinal epithelial proliferation and apoptosis through decreasing exosomal miR-125a/b, miR-125a inhibitor-loaded primary intestinal myofibroblasts exosomes or NC inhibitor-loaded primary intestinal myofibroblasts exosomes were delivered to SBS rats. Histological results indicated that miR-125a inhibitor-loaded IMF exosomes recapitulated the proliferation-promoting and anti-apoptosis effects of GLP-2. Taken together, these results suggest that GLP-2 exerted its intestinotrophic functions by decreasing exosomes-loaded miR-125a/b and thus promoting the restoration of MCL1 expression *in vivo*. Our study therefore sheds new light on the application of GLP-2 treated intestinal exosomes or IMF exosomes as a potential therapeutic tool for short bowel syndrome or chronic intestinal failure. Exosomes therapy might be of significant advantages as the yield of exosomes from intestine is high and targeting specificity to intestinal epithelial cells is relatively high due to the surface protein profile.

The present study has several limitations. Firstly, although our data suggested that GLP-2 mediated proliferation and apoptosis of HIEC6 through decreasing miR-125a/b contents in exosomes secreted by intestinal myofibroblasts, more detailed studies are necessary to identify GLP-2 induced any other changes in exosomes secreted by other cells in intestine tissue. Second, intestinal exosomes contain a multitude of bioactive molecules, not only miRNA. Many of the other components undoubtedly are bioactive, exerting the overall functional benefits as an ensemble. Whether any additional effectors or miRNAs are involved in the GLP-2-regulated growth of intestinal mucosa has not been elaborated and requires further investigation.

## Conclusions

This study demonstrated the critical role of exosomal miR-125a/b in GLP-2 mediated proliferation-promoting and apoptosis-preventing effects on intestinal epithelial cell *in vivo* and vitro, revealing a novel mechanism of genetic exchange between cells in the intestinal microenvironment. miR-125a/b inhibited the proliferation and promotes apoptosis of intestinal epithelial cells by suppressing MCL1. Exosomes therapy might be a potential therapeutic tool for short bowel syndrome or chronic intestinal failure in the future.

## Supplementary Material

Supplementary figures and tables.

## Abbreviations

GLP-2: glucagon-like peptide-2
SBS: short bowel syndrome
NTA: nanoparticle trafficking analysis
MCL1: myeloid cell leukemia-1
3'-UTR: 3'-untranslated region
CIF: chronic intestinal failure
PN: parenteral nutrition
GLP-2R: glucagon-like peptide-2 receptor
ISEMFs: intestinal subepithelial myofibroblasts
PBS: phosphate buffer solution
HE: hematoxylin and eosin
TUNEL: terminal-deoxynucleoitidyl transferase mediated nick end labeling
DMEM: dulbecco's modified eagle medium
HIEC6: human intestinal epithelial cell
NC: negative control
FITC: fluorescein isothiocyanate
GAPDH: glyceraldehyde-3-phosphate dehydrogenase
qPCR: quantitative polymerase chain reaction
GLP-1: glucagon-like peptide-1
PCNA: proliferating cell nuclear antigen
FATP4: fatty acid transport protein 4
IGF-1R: insulin-like growth factor 1 receptor
SGLT1: sodium-glucose co-transporter 1
CCK-8: cell counting kit-8
IMF: intestinal myofibroblasts
GPCR: g protein-coupled receptor
EGF: epidermal growth factor
