# Supplementary material for: Exosomes-mediated Transfer of miR-125a/b in Cell-to-cell Communication: A Novel Mechanism of Genetic Exchange in the Intestinal Microenvironment
Source: Theranostics. 2020 Jun 12;10(17):7561–80. doi: 10.7150/thno.41802 (PMC7359098; doi:10.7150/thno.41802)
Supplement: Supplementary file 1 — Supplementary figures and tables. [file thnov10p7561s1.pdf]

Supplementary Methods

Plasma GLP-1 and GLP-2 Measurements

Blood samples were collected by heart puncture into ice-chilled tubes containing EDTA (3.7 mmol/L), diprotin A (0.1 mmol/L; Sigma-Aldrich, USA) and aprotinin (0.01 mmol/L; Calbiochem, CA) to avoid GLP-2 degradation. The tubes were immediately shaken and centrifuged at 4 °C for 15 minutes. Plasma was collected and stored at −80 °C for further use. Plasma GLP-1 and GLP-2 were measured using corresponding ELISA kit according to the manufacturer's instructions (Cusabio Biotech Company, China).

miRNA library construction and sequencing

miRNA library preparation and sequencing were conducted by a commercial service (Huayin health, China). Briefly, total RNAs were extracted from 300 ug intestinal exosomes. Both 3' and 5' adaptors were added to each end, respectively, followed by reverse transcription and polymerase chain reaction (PCR) amplification. The PCR products derived from the 18-30 nucleotide RNA molecules were purified by electrophoresis and sequenced using the Illumina HiSeq 2500 platform.

Cell isolation and culture

Primary intestinal myofibroblasts (IMF) were isolated from rat intestinal mucosa based on previously described methods [1, 2]. Mucosal sheets were treated with 1 mM EDTA in Ca^{2+} free HBSS buffer three times at 37 °C for 30 min with gentle rocking at 80 cycles/min to remove the epithelial layer. The mucosal sheet without epithelial cells was then cut into small pieces and cultured in DMEM containing 10% FBS, 1% antimycotic/antibiotic mix, glutamine containing 75 U/mL collagenase II (Sigma), 125 μg/mL collagenase and dispase mix (Roche) and then incubated for 2 h at 37 °C. Supernatant with single cells was centrifuged at 300 g for 5 min, washed twice with PBS and cultured in DMEM containing 10% FBS and 1% antimycotic/antibiotic at 37 °C with 5% CO_2. The cultured IMF were used for experiments after three to five passages.

Two cell lines HIEC6 (CRL-3266) and GLP-2 receptor-positive CCD-18Co (CRL-1459) were obtained from American Type Culture Collection (ATCC, USA). HIEC6 Cells were cultured in OptiMEM (Gibco, USA) supplemented with 4% fetal bovine serum (FBS, Gibco), 1% penicillin-streptomycin, 20 mM HEPES, 10 mM GlutaMAX and 10 ng/mL epidermal growth factor (EGF). CCD-18Co cells were cultured in EMEM supplemented with 10% FBS and 1% penicillin-streptomycin. The cultures were maintained at 37 °C in 5% CO_2 and 95% humidity.

Mouse organoids culture

Intestinal crypts from C57B/6J mice were isolated according to the manufacturer's instructions of STEMCELL Technologies and previous published study [3]. Approximately 1500 crypts were embedded into phenol red-free Matrigel (Corning, 20 ul/well in 48-well plates) and cultured in complete IntestiCult™ Organoid Growth Medium (Stem cell, 200 μL/well).Organoids were removed from Matrigel in every 5-7 days, mechanically disrupted by pipetting, centrifuged at 300 g for 5 min and they were then embedded into new Matrigel. 20 μg IMF-Exo were added to each well with organoids.

RNA isolation and quantitative real-time PCR

Total RNA was extracted from jejunum mucosal scrapings, exosomes or cells using TRIzol reagent (Invitrogen, USA) following the standard procedures. The concentration and purity of the RNA were determined using a NanoDrop Spectrophotometer. To detect mature miR-125a/b-5p, specific TaqMan miRNA Assay Probes (Taqman microRNA Assays, 002198 & 000449,

ThermoFisher Scientific, USA) were used. U6 snRNA served as an internal control. To assess gene expression, RNA was reversely transcribed into cDNA using a HiScript II 1st Strand cDNA Synthesis Kit (Vazyme) and quantitative real-time PCR (qRT-PCR) was performed using SYBR Green dye. The sequences of the primers are shown in Supplemental Table 1.

Supplemental Table 1

Gene symbol	Species	Primer sequences	
		F (sense)	R (antisense)
GAPDH	Homo	CGAGCCACATCGCTCAGACA	GTGGTGAAGACGCCAGTGGA
Bcl-2	Homo	TGTGTGTGGAAGAGCGTCAAC	CCCAGACTCACATCACCAAGT
Bax	Homo	GGGGAGCAGCCCAGAGG	CACTCGCTCAGCTTCTTGGT
PCNA	Homo	AGCCTGACAAATGCTTGCTG	TCTAGCTGGTTTCGGCTTCAG
Ki67	Homo	CGTCCCAGTGGAAGAGTTGT	CGACCCCGCTCCTTTTGATA
IGF-1R	Homo	GGCTGGGGCTCTTGTTTACC	CTCTCTCGAGTTCGCCTGGT
SGLT1	Homo	CTGCCACCATGGACAGTAGC	GGCCATCACTACCACGAAGT
FATP4	Homo	ACCCGCCTCATCTGTTCATT	TGCCAGATTCACATGGGCAC
MCL-1	Homo	AACGCGGTAATCGGACTCAA	CCTCCTTCTCCGTAGCCAAA

Protein isolation and western blot

RIPA lysis buffer (Beyotime, China) with 1% PMSF (Beyotime, China) and 1% protease inhibitor (Thermo scientific, USA) was used to isolate protein from tissues or cells. Proteins were separated by 10% SDS-PAGE, transferred onto polyvinylidene difluoride membranes ((Millipore). Membranes were blocked with 5% bovine serum albumin and incubated with primary antibodies targeting the following proteins: Caspase3, MCL-1, CD9, Alix, TSG101, Rab 27a, and nSMase2 (Abcam, USA); CD63 (SBI, USA); Rab 27b (Merck, USA); PCNA and Ki67 (Cell Signaling Technology, USA) and GAPDH (Bioworld, USA). After binding with corresponding secondary antibodies, protein bands were visualized using enhanced chemiluminescence (ECL).

References

1. Islam MS, Kusakabe M, Horiguchi K, Iino S, Nakamura T, Iwanaga K, et al. PDGF and TGF-beta promote tenascin-C expression in subepithelial myofibroblasts and contribute to intestinal mucosal protection in mice. Br J Pharmacol. 2014; 171: 375-88.

2. Leen JL, Izzo A, Upadhyay C, Rowland KJ, Dube PE, Gu S, et al. Mechanism of action of glucagon-like peptide-2 to increase IGF-I mRNA in intestinal subepithelial fibroblasts.

Endocrinology. 2011; 152: 436-46.

3. Oszvald A, Szvicsek Z, Sandor GO, Kelemen A, Soos AA, Paloczi K, et al. Extracellular vesicles transmit epithelial growth factor activity in the intestinal stem cell niche. Stem Cells. 2020; 38: 291-300.

Supplementary Figures

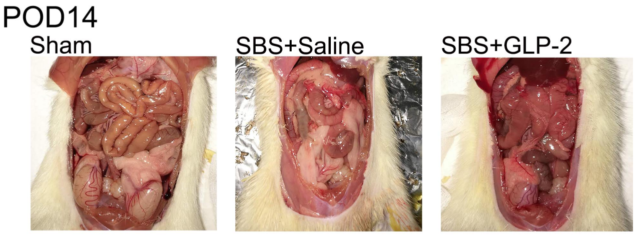

Figure S1. Gross morphology of the residual jejunum at 14 days post-operation.

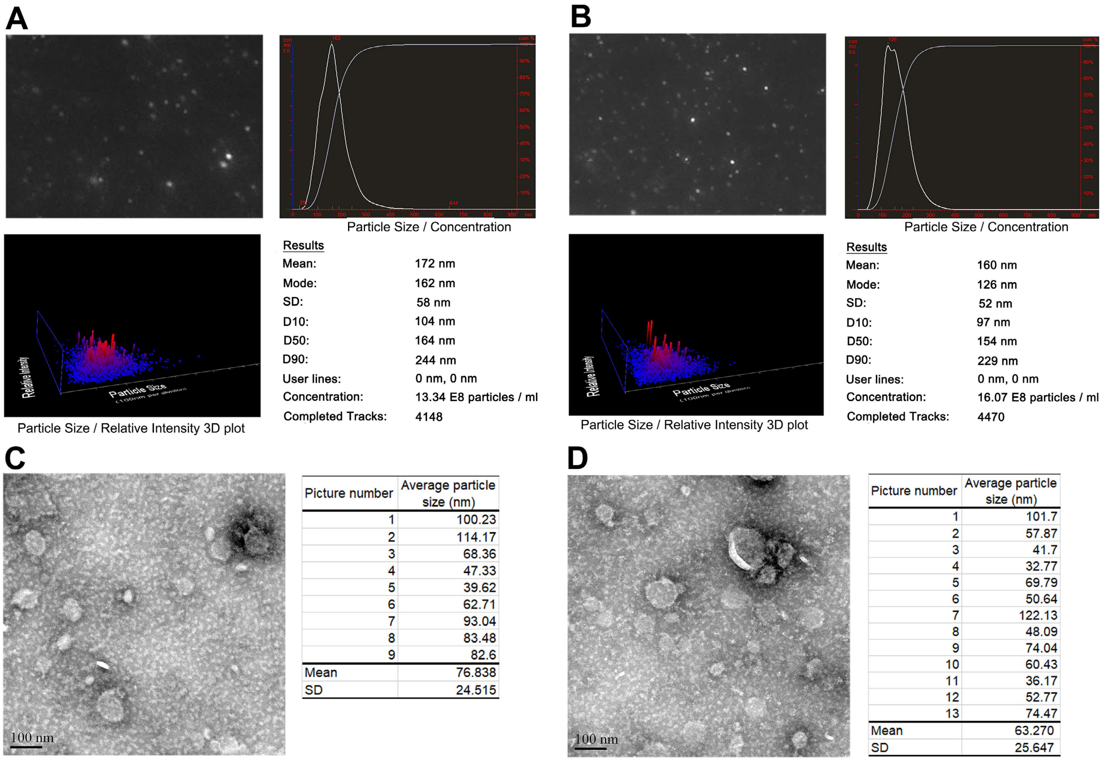

Picture number	Average particle size (nm)
1	100.23
2	114.17
3	68.36
4	47.33
5	39.62
6	62.71
7	93.04
8	83.48
9	82.6
Mean	76.838
SD	24.515

Picture number	Average particle size (nm)
1	101.7
2	57.87
3	41.7
4	32.77
5	69.79
6	50.64
7	122.13
8	48.09
9	74.04
10	60.43
11	36.17
12	52.77
13	74.47
Mean	63.270
SD	25.647

Figure S2. Identification of SBS-Exo and GLP2-Exo. (A-B) Nanoparticle trafficking analyzed the diameters and concentration of SBS-Exo and GLP2-Exo. (C-D) Transmission electron micrograph and particle size distribution of SBS-Exo and GLP2-Exo.

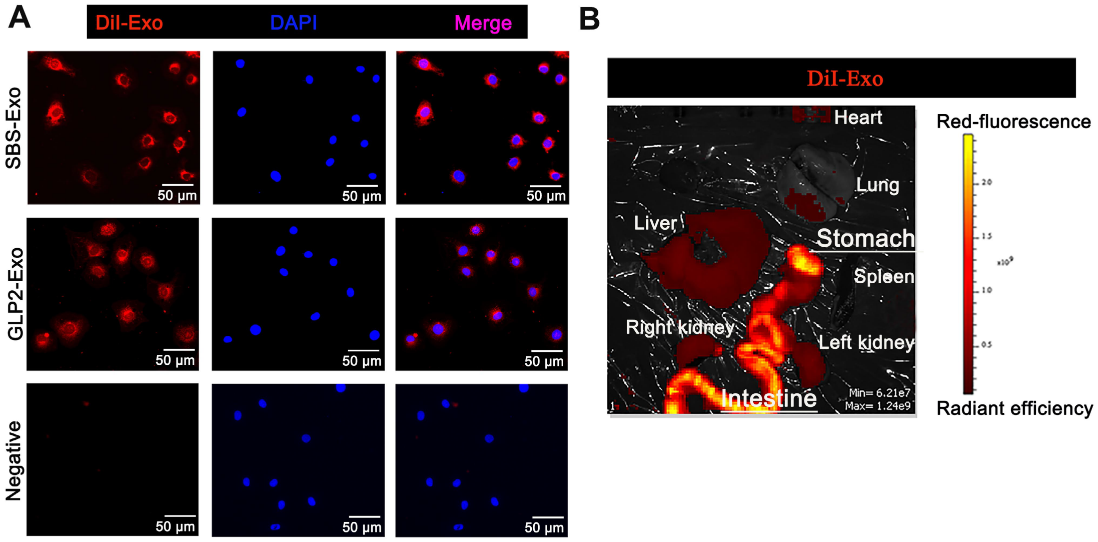

Figure S3. In vitro and in vivo uptake of intestinal exosomes. (A) The DiI (red)-labeled intestinal exosomes (SBS-Exo and GLP2-Exo) were internalized into intestinal epithelial cell (HIEC6). Negative control sample, aliquots isolated from equal volume of DMEM culture medium in the absence of enzymatically digested intestinal fragments were stained with DiI. (B) The DiI (red)-labeled intestinal exosomes were absorbed by various organs, especially the gastrointestinal tract at 24 h post intraperitoneal injection. Absorption efficiency was analyzed using Living Image 3.1 software.

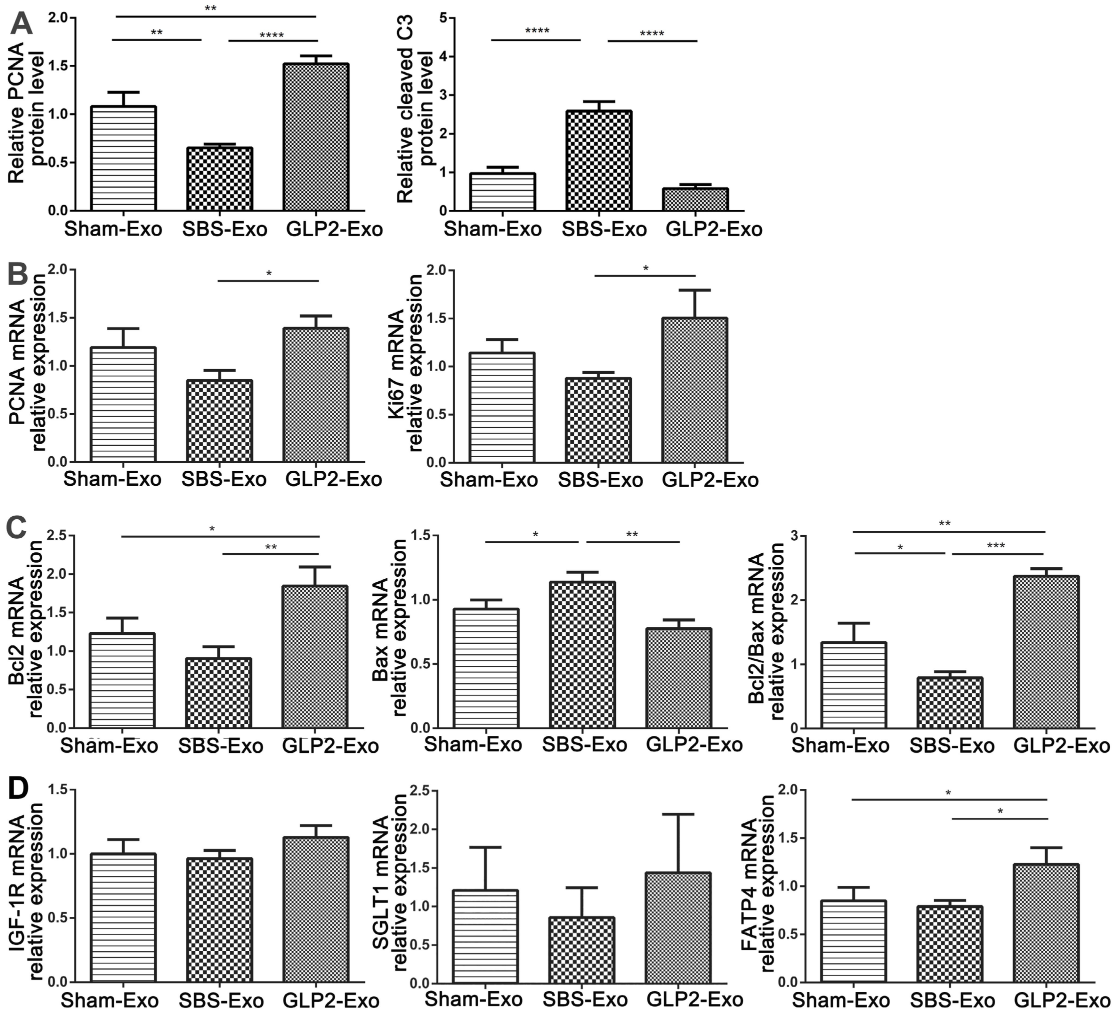

Figure S4. Protein and mRNA level quantification of HIEC6 cultured with Sham-Exo, SBS-Exo or GLP2-Exo. (A) Quantitative analysis of Western blots for PCNA and Cleaved Caspase3 in Figure 3E. (B) Gene expression profiles of proliferation marker PCNA and Ki67 in HIEC6 after culturing with Sham-Exo, SBS-Exo or GLP2-Exo for 48 h. (C) Gene expression profiles of anti-apoptotic Bcl-2 and pro-apoptotic Bax in HIEC6 treated with equal doses of different kinds of intestinal exosomes. (D) Gene expression profiles of insulin-like growth factor 1 receptor (IGF-1R) and nutrient transporters (Sodium-glucose co-transporter 1, SGLT1; Fatty acid transport protein 4, FATP4) in HIEC6 treated with intestinal exosomes. N=3, *p < 0.05, **p < 0.01, ***p < 0.001, and ****p < 0.0001.

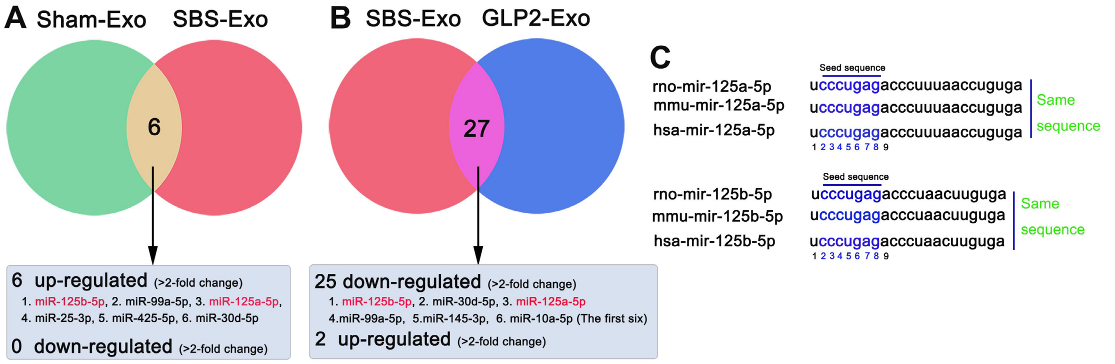

Figure S5. miRNA profiling assays in Sham-Exo, SBS-Exo and GLP2-Exo. (A) Changes of miRNA levels in SBS-Exo as compared with Sham-Exo. (B) Changes of miRNA levels in GLP2-Exo as compared with SBS-Exo. (C) The sequences of miR-125a-5p and miR-125b-5p in rat, mouse and human. The seed sequences (nucleotides 2-8$^{5'\rightarrow 3'}$) of miR-125a-5p and miR-125b-5p are marked in red.

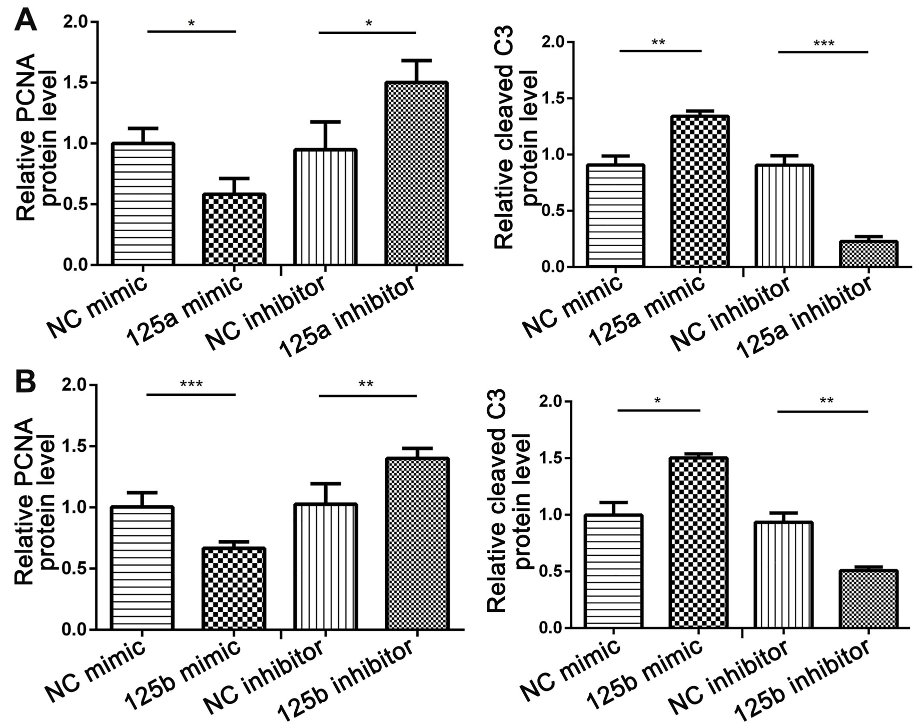

Figure S6. Protein level quantification of HIEC6 transfected with NC mimic, miR-125a/b mimic, NC inhibitor or miR-125a/b inhibitor. (A-B) Quantitative analysis of Western blots for PCNA and Cleaved Caspase3 protein levels in Figure 4K. N=3, *p < 0.05, **p < 0.01, and ***p < 0.001.

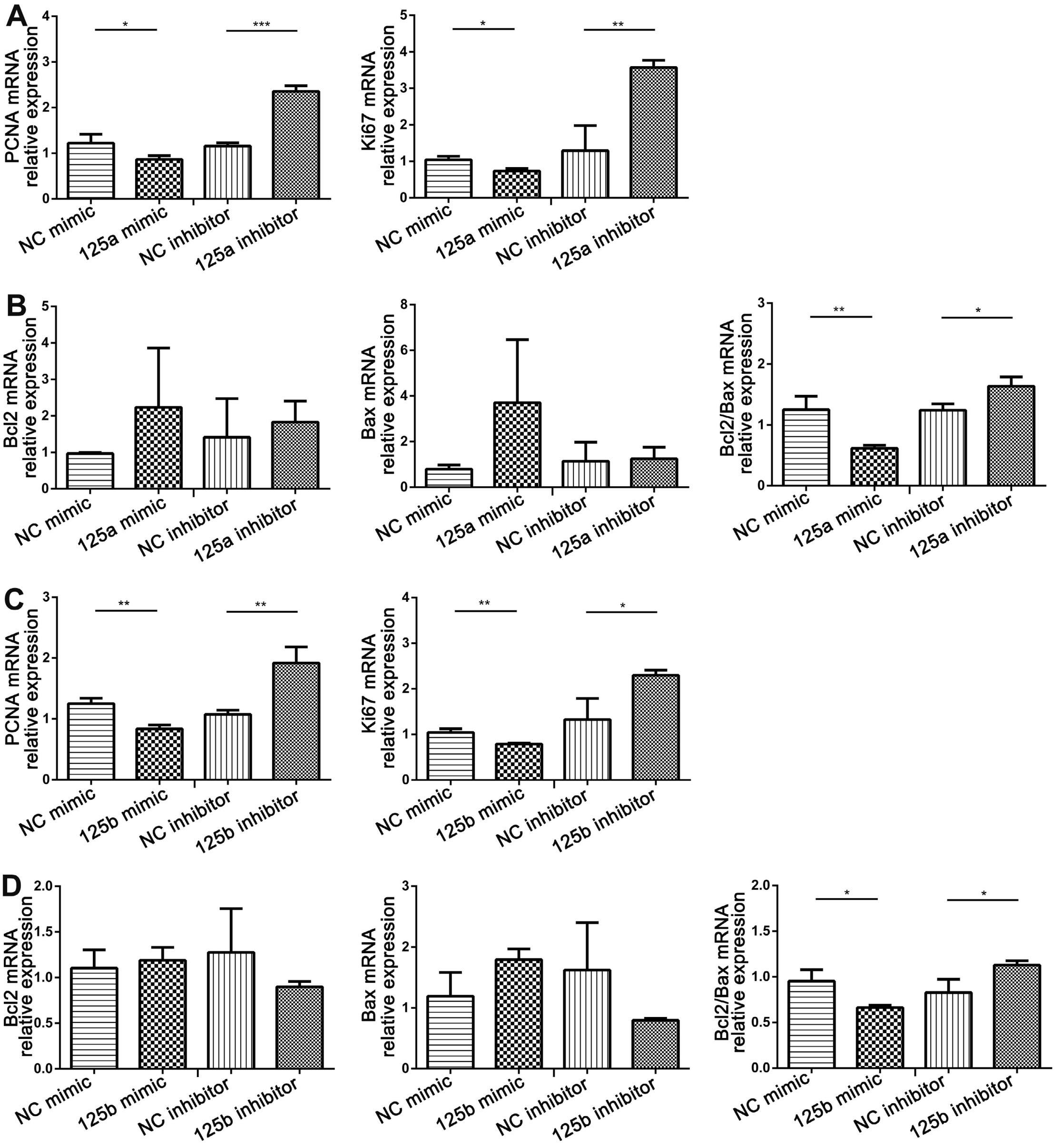

Figure S7. mRNA level quantification of HIEC6 transfected with NC mimic, miR-125a/b mimic, NC inhibitor or miR-125a/b inhibitor. (A) Gene expression profiles of proliferation marker PCNA and Ki67 in HIEC6 transfected with equal doses of NC mimic, miR-125a mimic, NC inhibitor or miR-125a inhibitor. (B) Gene expression profiles of anti-apoptotic Bcl-2 and pro-apoptotic Bax in HIEC6 transfected with equal doses of NC mimic, miR-125a mimic, NC inhibitor or miR-125a inhibitor. (C) Quantitative analysis of PCNA and Ki67 mRNA levels in HIEC6 transfected with equal doses of NC mimic, miR-125b mimic, NC inhibitor or miR-125b inhibitor. (D) Quantitative analysis of anti-apoptotic Bcl-2 and pro-apoptotic Bax mRNA levels in HIEC6 transfected with equal doses of NC mimic, miR-125b mimic, NC inhibitor or miR-125b inhibitor. N=3, $*p < 0.05$, $**p < 0.01$, and $***p < 0.001$.

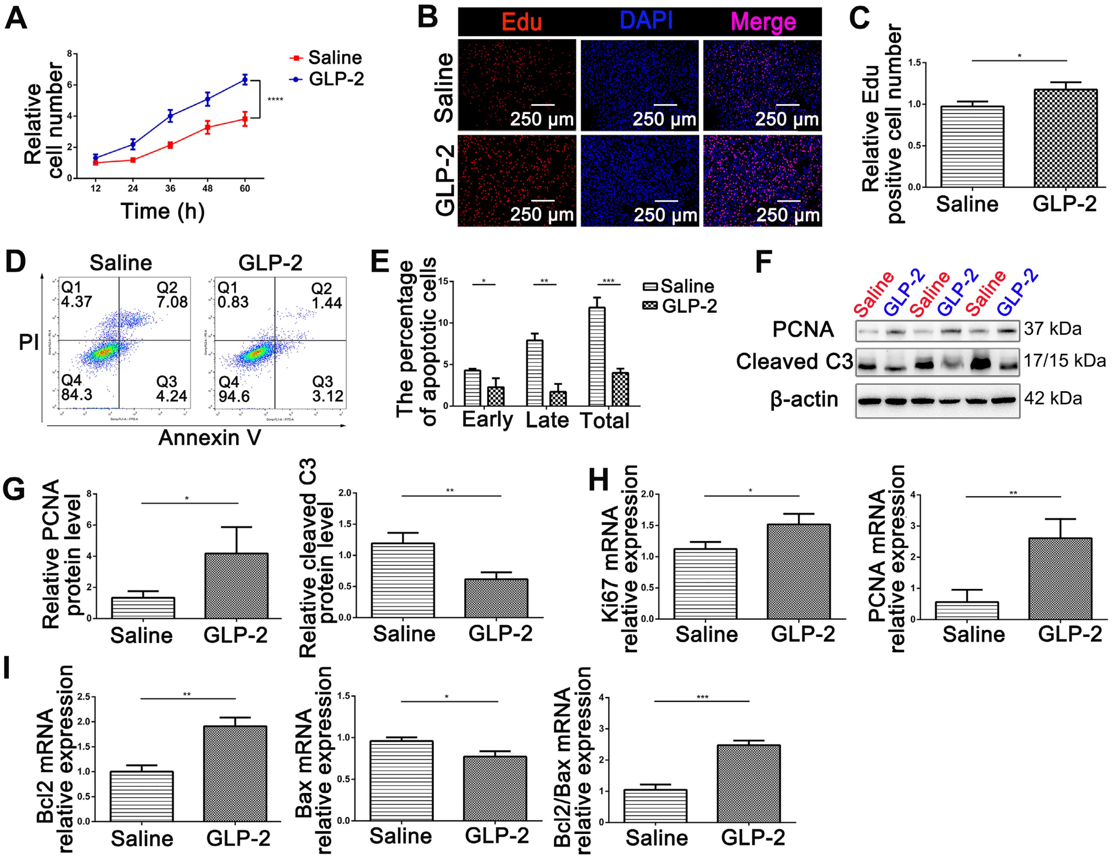

Figure S8. GLP-2 modulated the proliferation and apoptosis of CCD-18Co cells. (A) CCK-8 were performed 12, 24, 36, 48 and 60 h after adding 0.1 μg/mL GLP-2 or vehicle to CCD-18Co cells. (B) Representative images of EdU staining in HIEC6 with or without GLP-2 treatment. (C) Quantitative assessment of percentage of EdU positive cells in (B). (D) Representative flow cytometry plots showing the percentages of early apoptotic cells (Annexin V^{+}/PI^{-}), late apoptotic cells (Annexin V^{+}/PI^{+}) and total (early+late) apoptotic cells in HIEC6 with or without GLP-2 treatment. (E) Pooled flow cytometry data from (D). (F) Western blot assay for PCNA and Cleaved C3 expression in CCD-18Co cells under saline or GLP-2 intervention. (G) Quantitative analysis of PCNA and Cleaved C3 levels in (F). (H) Quantitative analysis of PCNA and Ki67 mRNA levels in CCD-18Co cells with or without GLP-2 treatment. (I) Quantitative analysis of anti-apoptotic Bcl-2 and pro-apoptotic Bax mRNA levels in CCD-18Co cells with or without GLP-2 treatment. N=3, *p < 0.05, **p < 0.01, ***p < 0.001, and ****p < 0.0001.

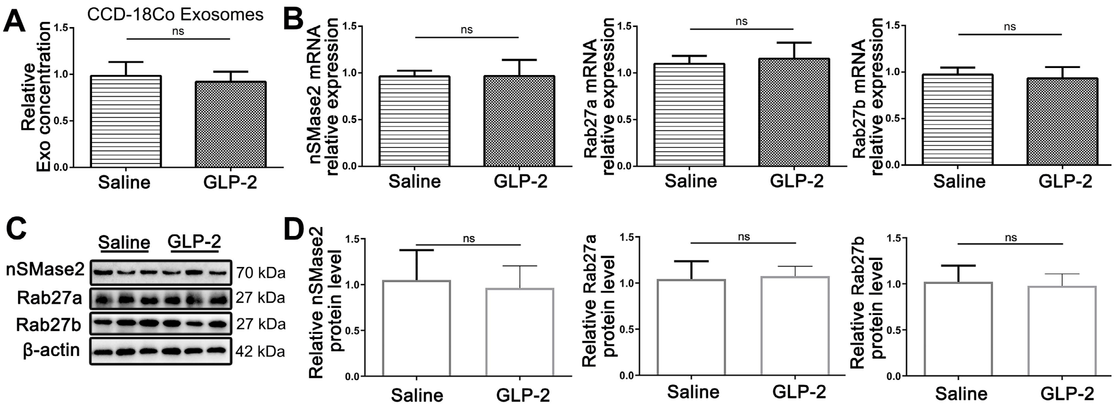

Figure S9. GLP-2 do not affect the packaging and release of exosomes in CCD-18Co cells. (A)

Relative concentration of exosomes derived from saline or GLP-2 treated CCD-18Co cells detected by NTA. (B) Gene expression profiles of critical mediators of exosomes packaging and secretion nSMase2, Rab27a and Rab27b in CCD-18Co with or without GLP-2 treatment. (C) Representative blots of nSMase2, Rab27a and Rab27b protein levels in CCD-18Co with or without GLP-2 treatment. (D) Pooled data from immunoblots in (C). N=3, ns=no significant.

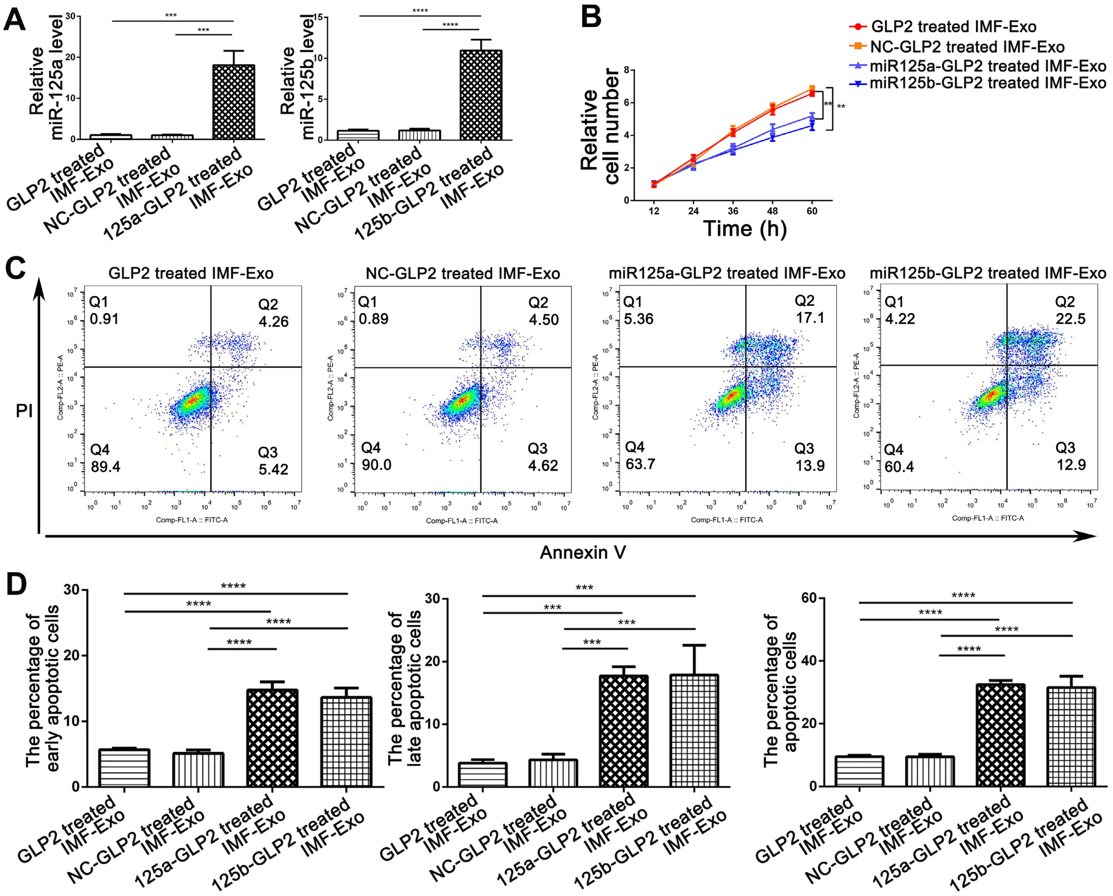

Figure S10. The proliferative and anti-apoptotic effects of GLP-2 treated IMF-Exo were negated by increasing miR-125a/b contents in GLP-2 treated IMF-Exo. (A) qPCR analysis of miR-125a/b levels in miR-125a/b mimic-loaded GLP-2 treated IMF-Exo, NC mimic-loaded GLP-2 treated IMF-Exo and GLP-2 treated IMF-Exo. (B) CCK-8 were performed 12, 24, 36, 48 and 60 h after co-culture of HIEC6 with equal doses of miR-125a/b mimic-loaded GLP-2 treated IMF-Exo, NC mimic-loaded GLP-2 treated IMF-Exo and GLP-2 treated IMF-Exo. (C) Representative flow cytometry plots showing the percentages of early apoptotic cells (Annexin V^+/PI^-), late apoptotic cells (Annexin V^+/PI^+) and total (early+late) apoptotic cells in HIEC6 after culturing with miR-125a/b mimic-loaded GLP-2 treated IMF-Exo, NC mimic-loaded GLP-2 treated IMF-Exo and GLP-2 treated IMF-Exo. (D) Pooled flow cytometry data from (C). N=3, $^{**}p < 0.01$, $^{***}p < 0.001$, and $^{****}p < 0.0001$.

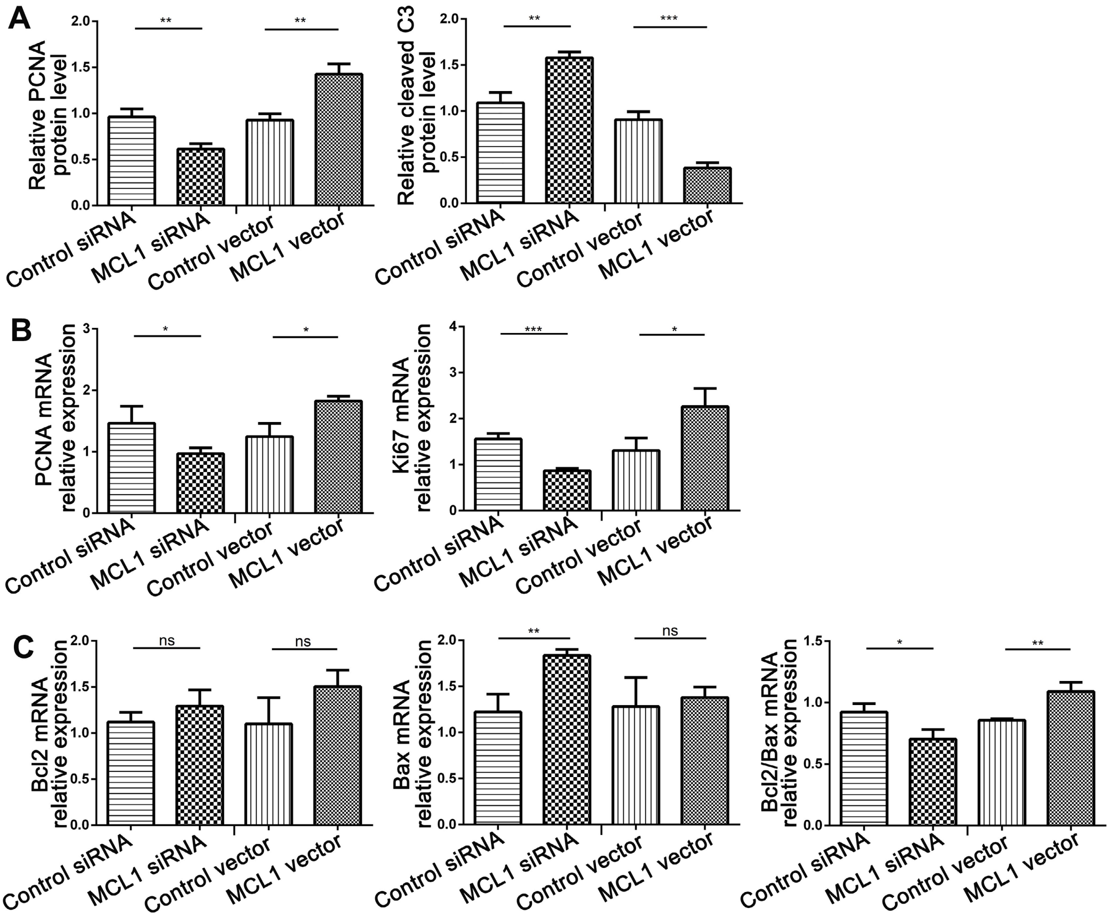

Figure S11. Protein and mRNA level quantification of HIEC6 intervened by MCL1 siRNA or MCL1 plasmid. (A) Quantitative analysis of Western blots for PCNA and Cleaved Caspase3 in Figure 8I. (B) Gene expression profiles of proliferation marker PCNA and Ki67 in HIEC6 transfected with MCL1 siRNA, Control siRNA, MCL1 plasmid, or Control plasmid. (C) Gene expression profiles of anti-apoptotic Bcl-2 and pro-apoptotic Bax in HIEC6 transfected with MCL1 siRNA, Control siRNA, MCL1 plasmid, or Control plasmid. N=3, *p < 0.05, **p < 0.01, and ***p < 0.001.

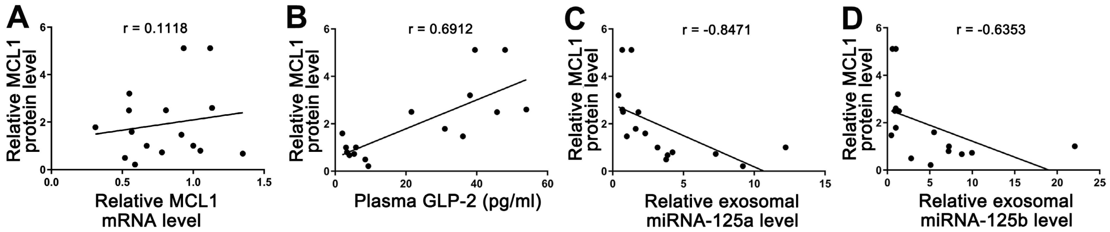

Figure S12. Correlation of relative MCL1 protein levels in the jejunum mucosa of SBS rats with MCL1 mRNA level (A), plasma GLP-2 concentration (B), intestinal exosomal miR125a level (C) and intestinal exosomal miR125b level (D). N=16.

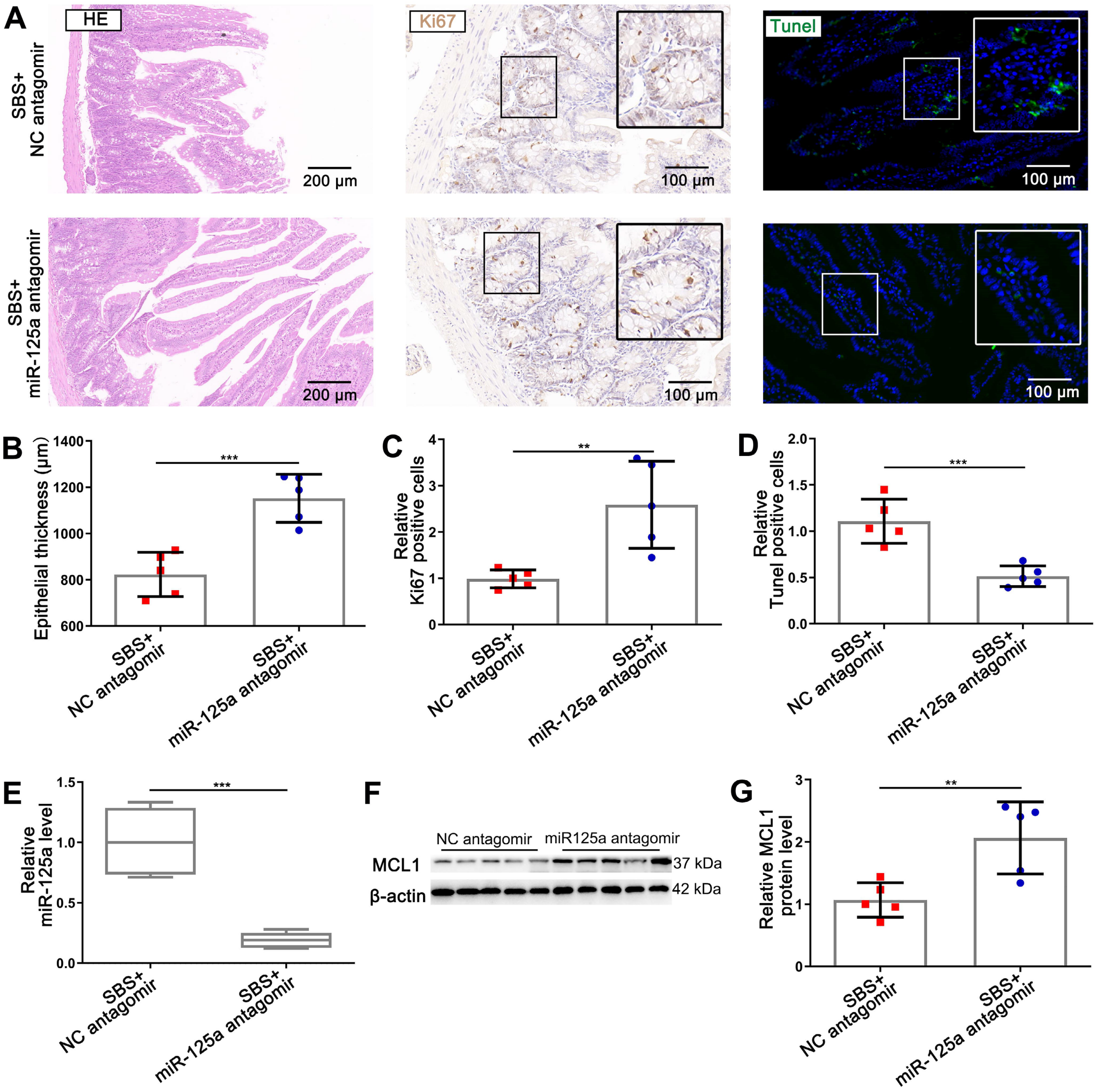

Figure S13. miR-125a antagomir enhanced intestinal epithelial proliferation and reduced intestinal epithelial apoptosis in vivo by rescuing the suppression of MCL1. (A) Representative images of H&E staining, Ki67 staining and TUNEL staining of remaining jejunum in SBS rats treated with miR-125a antagomir or NC antagomir. (B) Quantification of intestinal epithelial thickness in (A). (C) Quantitative analysis of percentage of Ki67 positive cells in (A). (D) Quantitative analysis of percentage of TUNEL positive cells in (A). (E) qPCR analysis of miR-125a level in jejunum mucosa of SBS rats treated with miR-125a antagomir or NC antagomir. (F) Representative blots of MCL1 levels in jejunum mucosa of SBS rats treated with miR-125a antagomir or NC antagomir. (G) Quantification of MCL1 protein levels in jejunum mucosa of SBS rats treated with miR-125a antagomir or NC antagomir. N=5, **$p < 0.01$, ***$p < 0.001$.
